# Toward robust lithium–sulfur batteries *via* advancing Li_2_S deposition†

**DOI:** 10.1039/d4sc02420f

**Published:** 2024-05-01

**Authors:** Xun Jiao, Xiaoxia Tang, Jinrui Li, Yujiao Xiang, Cunpu Li, Cheng Tong, Minhua Shao, Zidong Wei

**Affiliations:** a State Key Laboratory of Advanced Chemical Power Sources, School of Chemistry and Chemical Engineering China lcp@cqu.edu.cn tongcheng@cqu.edu.cn zdwei@cqu.edu.cn; b Department of Chemical and Biological Engineering, The Hong Kong University of Science and Technology Clear Water Bay Kowloon Hong Kong

## Abstract

Lithium–sulfur batteries (LSBs) with two typical platforms during discharge are prone to the formation of soluble lithium polysulfides (LiPS), leading to a decrease in the cycling life of the battery. Under practical working conditions, the transformation of S_8_ into Li_2_S is cross-executed rather than a stepwise reaction, where the liquid LiPS to solid Li_2_S conversion can occur at a high state of charge (SOC) to maintain the current requirement. Therefore, advancing Li_2_S deposition can effectively reduce the accumulation of LiPSs and ultimately improve the reaction kinetics. Herein, a “butterfly material” GeS_2_-MoS_2_/rGO is used as a sulfur host. Rich catalytic heterointerfaces can be obtained *via* the abundant S–S bonds formed between GeS_2_ and MoS_2_. MoS_2_ (left wing) can enhance LiPS adsorption, while the lattice-matching nature of *Fdd*2 GeS_2_ (right wing) and *Fm*3̄*m* Li_2_S can induce multiple nucleation and regulate the 3D growth of Li_2_S. Li_2_S deposition can be advanced to occur at 80% SOC, thereby effectively inhibiting the accumulation of soluble LiPSs. Attributed to the synergistic effect of catalytic and lattice-matching properties, robust coin and pouch LSBs can be achieved.

## Abstract 2

The GeS_2_-MoS_2_/rGO heterostructure is designed as a sulfur host to facilitate the conversion of polysulfides and advance the deposition of Li_2_S synergistically by strengthening the hybrid current during cycling.

## Graphical abstract

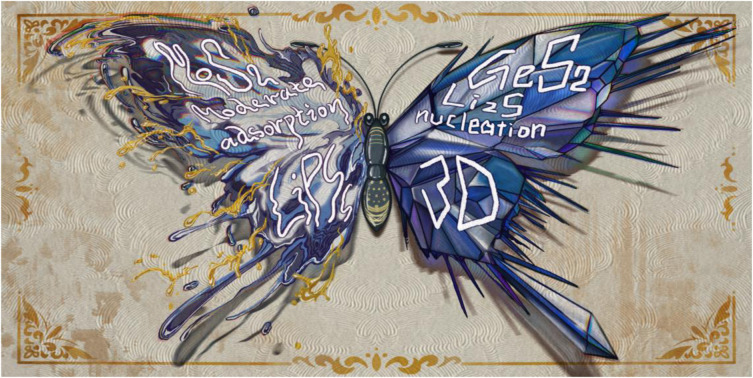

## 1 Introduction

**Scheme 1:**
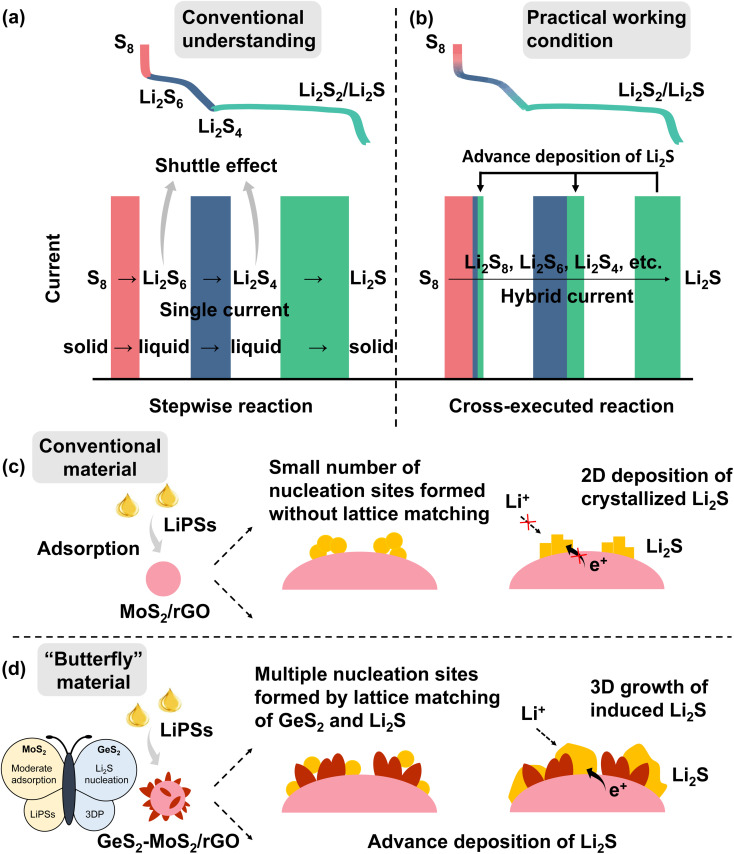
Conventional understanding (a) and practical working conditions (b) of discharge of LSBs (the red line and rectangle are regarded as solid–liquid reactions from S_8_ to Li_2_S_6_, the blue line and rectangle are regarded as liquid–liquid reactions from Li_2_S_6_ to Li_2_S_4_, and the green line and rectangle are regarded as liquid–solid reactions from Li_2_S_4_ to Li_2_S). In contrast to the conventional understanding of a stepwise reaction (a), the practice cycling condition is in fact a cross-executed reaction (b). Strengthening the hybrid current during cycling of LSBs can promote the adsorption and conversion of sulfur species and ultimately enhance the redox kinetics of the batteries; (c and d) schematic illustration of LiPS conversion and Li_2_S growth on the MoS_2_/rGO surface (c) and GeS_2_-MoS_2_/rGO surface (d). (d) The designed “butterfly material”: MoS_2_ (left wing) can enhance LiPS adsorption, while the lattice-matching nature of *Fdd*2 GeS_2_ (right wing) and *Fm*3̄*m* Li_2_S can induce multiple nucleation and regulate the 3D growth of Li_2_S. The wings of the butterfly enable advanced deposition of Li_2_S.

As a promising alternative to lithium-ion batteries (LIBs), lithium–sulfur batteries (LSBs) have attracted widespread attention with their theoretical energy density of more than 2600 W h kg^−1^, as well as the eco-friendliness and low cost of sulfur.^1–5^ According to conventional understanding, the discharge of sulfur species is a stepwise reaction (Scheme 1(a)).^6,7^ During the discharge process, the reaction of sulfur species first undergoes a phase transition from solid S_8_ to liquid long-chain lithium polysulfides (LiPSs, Li_2_S_*x*_, 6 ≤ *x* ≤ 8) (red line and rectangle in Scheme 1(a)), then to short-chain Li_2_S_4_ (blue line and rectangle in Scheme 1(a)), and finally to solid Li_2_S_2_/Li_2_S (green line and rectangle in Scheme 1(a)), which is called the “solid–liquid–solid” conversion mechanism.^8–10^ Throughout the discharge process, the accumulated soluble LiPSs will dissolve in the electrolyte, causing the loss of active material, which is known as the “shuttle effect”.^11–13^ However, in fact, under practical discharge working conditions, the conversion reactions of different sulfur species are cross-executed rather than stepwise (Scheme 1(b)).

During the discharge process, when the preceding reactions cannot meet the current demand, the subsequent reactions will participate in the electrochemical reaction, thus forming a “hybrid current”.^14^ For example at 0.1 A, the number of electrons transferred on the electrode per second is determined (0.1/Faraday constant). During the discharge process of LSBs, the conversion of Li_2_S_6_ to Li_2_S_4_ cannot provide enough electrons in the electrode reaction, and the subsequent reaction of Li_2_S_4_ to Li_2_S_2_/Li_2_S will be involved simultaneously. So, the practical discharge/charge process produces a hybrid current, not a single current. The existence of hybrid current provides an opportunity to address the shuttle effect of LiPSs. If the adsorption of LiPSs is enhanced and then rapidly converted to Li_2_S_2_/Li_2_S, the subsequent liquid–solid reaction can be involved in the whole discharge process, which effectively reduces the accumulation of soluble LiPSs and greatly improves the redox kinetics of the battery. And the conversion of LiPSs to Li_2_S is the rate-determining step in the sulfur reduction reaction.^15^ Therefore, it is desirable to find a method to enhance the adoption of LiPSs and promote Li_2_S growth, and to advance the deposition of Li_2_S at a high state of charge (SOC). The advanced deposition of Li_2_S will effectively reduce the accumulation of liquid LiPSs, as well as facilitate the conversion of sulfur species.

According to many research studies, well-designed sulfur hosts with moderate adsorption ability and catalytic activity can alleviate the shuttle effect of LiPSs and improve reaction kinetics of LSBs.^16–18^ Whereas, it is known that the discharging product Li_2_S is an electronic–insulating ionic compound, which is difficult to deposit and grow rapidly on the substrate interface.^19–21^ In general, Li_2_S deposition begins with nucleation on the conductive substrate, and then increases at the interface of nucleation, substrate, and electrolyte.^22^ With the deposition and accumulation of Li_2_S, an insulating Li_2_S crystal structure is gradually formed on the conductive interface, leading to a gradual slowdown of Li_2_S growth, which ultimately limits the efficiency of electrochemical conversion in LSBs.^23,24^ Moreover, the first step, solid–solid decomposition of crystalline Li_2_S, produces an ultra-high overpotential.^25^ Therefore, developing the catalytic interconversion between LiPSs and Li_2_S, as well as improving the deposition and decomposition efficiency of Li_2_S, are particularly important towards the high performance LSBs.

In this regard, we designed and fabricated a “butterfly material” GeS_2_-MoS_2_/rGO. The nanosheet hierarchical petal-spherical GeS_2_-MoS_2_ heterostructure can enhance the reaction kinetics of LiPSs and advance the Li_2_S deposition. MoS_2_ (left wing) can enhance the LiPS adsorption, and GeS_2_ (right wing) can induce 3D Li_2_S deposition. And the introduction of reduced graphene oxide (rGO), a conductive carbon material, into the heterostructure can not only further enhance the electrical conductivity, but relieve the mechanical stress caused by the volume change of the electrode material during the cycling. The lattice-matching nature between orthorhombic GeS_2_ (*Fdd*2) and cubic Li_2_S (*Fm*3̄*m*) can guide Li_2_S growth in a 3D model, which reduces the Li_2_S transverse diffusion and avoids the catalyst surface passivation. What's more, the three-dimensional (3D) model deposited Li_2_S also ensures that the interface always provides channels for ionic and electronic conduction, exposing sufficient catalytically active sites for the conversion of Li_2_S.^26^ The wings of the butterfly promote Li_2_S growth and regulate the Li_2_S deposition behavior, and finally advance the Li_2_S formation at a high SOC (Scheme 1(c and d)). Therefore, robust LSBs with long-term cycling stability and potential for practical applications can be achieved.

## 2 Results and discussion

### 2.1 Material design

The heterostructure design combines the advantages of different components and provides a manipulable electronic structure, which is a good choice for promoting redox kinetics in LSBs.^27,28^ In the selection of catalysts, two-dimensional materials have been extensively studied in electrochemistry due to their unique physical and chemical properties.^29^ And molybdenum-based materials have been widely used in LIBs and LSBs due to their chemical stability and environmental friendliness.^30^ Among them, MoS_2_ is one of the most typical representatives of a tri-atomic layer (S–Mo–S) accumulated by weak van der Waals forces. MoS_2_ possesses abundant active sites, moderate adsorption ability toward LiPSs, and good electronic conductivity, which enables rapid liquid–liquid conversion and provides a high-speed electrochemical conversion pathway.^31^

More importantly, to improve sulfur utilization and redox kinetics, it is critical to introduce substrates capable of coordinating the Li_2_S deposition process: *Fdd*2 GeS_2_, and the *b*-axis of the lattice is equal to 22.67 Å, which is four times that of the typical reduction product of LSBs, *Fm*3̄*m* Li_2_S (*b* = 5.67 Å). The lattice mismatch (*f*) between *Fdd*2 GeS_2_ and *Fm*3̄*m* Li_2_S can be calculated from eqn (1):^32^

1 *f* = (*α*_s_ − *α*_g_)/*α*_s_

where *α*_s_ and *α*_g_ are the lattice constants of the substrate (GeS_2_) and the growth material (Li_2_S), respectively. In the *b*-axis direction, *α*_s_ (GeS_2_) is 22.67 Å, and *α*_g_ (Li_2_S) is 22.68 Å (5.67 × 4). As the *f* is significantly low (0.04%), when Li_2_S deposits onto GeS_2_, low mismatch dislocations and stress will be achieved, which further leads to low interfacial resistance and promotes electron transfer between the two phases.^33^ In the *a*-axis and *c*-axis directions, *α*_s_ (GeS_2_) is 6.87 Å, and *α*_g_ (Li_2_S) is 5.67 Å, respectively, so the *f* is calculated to be 17.47%. Moderate lattice mismatch (5–20%) may result in a lack of epitaxial correlation between the substrate and the growth material. What's more, because of the close match of lattice constants (*f* = 0.04%) in the *b*-axis direction, *Fm*3̄*m* Li_2_S can be readily grown on *Fdd*2 GeS_2_. That is, in the beginning, *Fm*3̄*m* Li_2_S selectively nucleates on one facet of the *Fdd*2 GeS_2_ substrate, and subsequently nucleates and grows on the other facets of *Fdd*2 GeS_2_, which ultimately produces multi-site deposition and 3D growth of Li_2_S. Therefore, the interface of the GeS_2_-MoS_2_ heterostructure always maintains a conductive network and Li^+^ transport channels as Li_2_S growth increases.

**Fig. 1:**
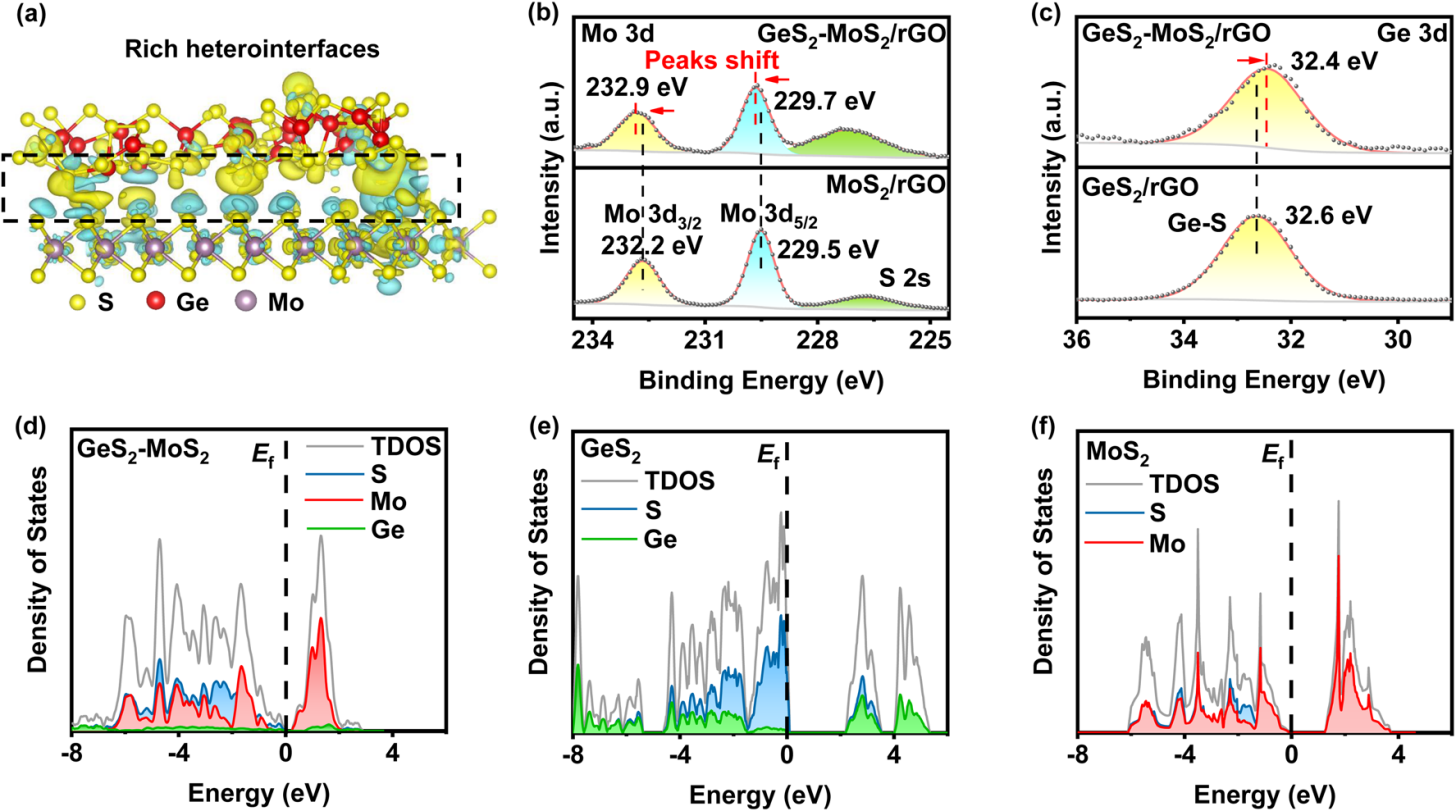
(a) Interfacial charge density difference of GeS_2_-MoS_2_ (yellow: electron accumulation; cyan: electron depletion). The strong bonding interactions of the heterostructure form rich catalytic heterointerfaces and multiple nucleation sites of Li_2_S. (b) High-resolution Mo 3d XPS spectra of GeS_2_-MoS_2_/rGO and MoS_2_/rGO. The Mo 3d peaks of GeS_2_-MoS_2_/rGO shift towards the higher binding energy region, compared with those for MoS_2_/rGO. (c) High-resolution Ge 3d XPS spectra of GeS_2_-MoS_2_/rGO and GeS_2_/rGO. Electron transfer from MoS_2_ to GeS_2_ in the GeS_2_-MoS_2_ heterostructure. (d–f) Calculated pDOS near the Fermi level of GeS_2_-MoS_2_, GeS_2_ and MoS_2_. GeS_2_-MoS_2_ possesses the smallest band gap, enhancing the conversion of LiPSs.

From the density functional theory (DFT) calculations, we can find that rich GeS_2_-MoS_2_ heterointerfaces can be constructed by the easily formed S–S bonds between GeS_2_ and MoS_2_. As displayed in Fig. 1(a), GeS_2_ (311) and MoS_2_ (002) planes are selected to construct the GeS_2_-MoS_2_ heterostructure. Abundant S–S bonds are formed between heterointerfaces, and the differential charge density diagram shows a significant charge accumulation and depletion at the interface of GeS_2_ and MoS_2_ in the heterostructure. And the interaction of rich catalytic heterointerfaces can favor intensive charge transfer. The charge transfer analysis is shown to allow charge redistribution at the GeS_2_-MoS_2_ interface, and it can be reasonably inferred that the GeS_2_-MoS_2_ heterostructure facilitates the interfacial charge transfer.

X-ray photoelectron spectroscopy (XPS) analysis was performed to experimentally confirm the interactions between GeS_2_ and MoS_2_ and study the chemical state of the different elements (Fig. 1(b, c) and S1†). The S 2p spectrum in Fig. S1(a)† shows two peaks at 163.8 and 162.7 eV, which correspond to S 2p_1/2_ and S 2p_3/2_ of S^2−^ species in GeS_2_-MoS_2_/rGO.^34^ In terms of the Mo 3d spectrum (Fig. 1(b)), the two major peaks at 232.9 eV (Mo 3d_3/2_) and 229.7 eV (Mo 3d_5/2_) of GeS_2_-MoS_2_/rGO are assigned to Mo^4+^ ions in MoS_2_.^35^ In the Ge 3d spectrum (Fig. 1(c)), the peak at 32.4 eV is a typical bonding of the Ge^4+^ ion in GeS_2_.^36^ More importantly, the Mo 3d spectrum of MoS_2_/rGO displays an obvious positive shift with the addition of GeS_2_, which is associated with electron transfer and strong interaction in the heterointerfaces, consistent with the interfacial charge arrangement predicted by DFT calculations.

Conductivity tests experimentally demonstrated the higher conductivity of GeS_2_-MoS_2_/rGO compared to GeS_2_/rGO and MoS_2_/rGO. Fig. S2† displays the current variations over 3000 s of testing at a constant voltage of 1.0 V to compare the conductivity of different catalysts. The electronic conductivity (*σ*) is calculated according to eqn (2) and (3):^37^2
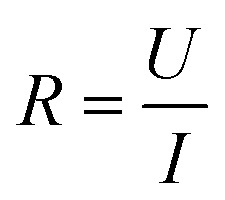
3
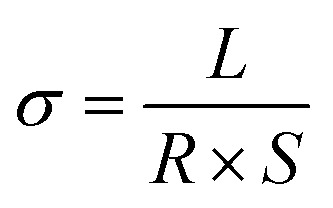


In eqn (2), *U* is the constant voltage (1.0 V), *I* is the average current (A) from 100 to 3000 s, and *R* is the calculated resistance (1/*S*). In eqn (3), *L* is the thickness of the sample (mm) and *S* is the area of the sample (132.665 mm^2^). The *σ* values of different catalysts are listed in Table S1.† And the result shows that GeS_2_-MoS_2_/rGO has the largest *σ* value, demonstrating the enhanced electrical conductivity of the heterostructure.

The projected densities of states (pDOSs) are then shown in Fig. 1(d–f) to assess the electronic structure differences of the different catalysts. All three catalysts have semiconductor properties, with the GeS_2_-MoS_2_ heterostructure showing the smallest band gap (≈ 0.19 eV), much smaller than that of GeS_2_ (≈ 2.14 eV) and MoS_2_ (≈ 1.21 eV). These results demonstrate that the GeS_2_-MoS_2_ heterostructure has good electrical conductivity and enhanced adsorption energies with LiPSs, which is attributed to the rich catalytic heterointerfaces and strong interfacial synergistic effect.

**Fig. 2:**
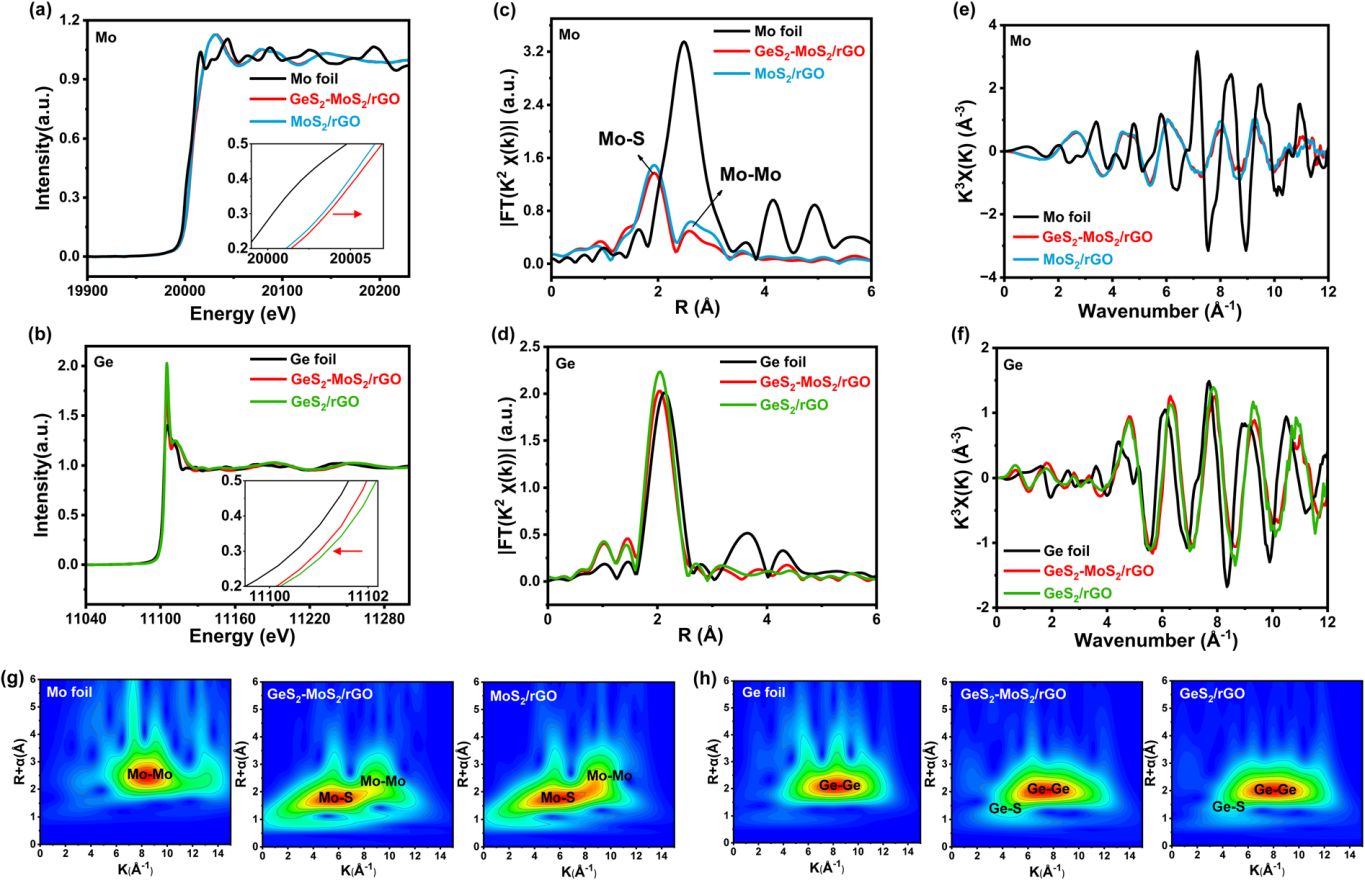
(a) Mo K-edge XANES spectra of Mo foil, GeS_2_-MoS_2_/rGO and MoS_2_/rGO. (b) Ge K-edge XANES spectra of Ge foil, GeS_2_-MoS_2_/rGO and GeS_2_/rGO. Electron transfer from MoS_2_ to GeS_2_ in the heterointerfaces. (c) *R*-space of EXAFS analysis of Mo in Mo foil, GeS_2_-MoS_2_/rGO and MoS_2_/rGO. (d) *R*-space of EXAFS analysis of Ge in Ge foil, GeS_2_-MoS_2_/rGO and GeS_2_/rGO. (e) Mo K-edge EXAFS oscillations of Mo foil, GeS_2_-MoS_2_/rGO andMoS_2_/rGO. (f) Ge K-edge EXAFS oscillations of Ge foil, GeS_2_-MoS_2_/rGO and GeS_2_/rGO. (g) WT contour plots at the Mo K-edge of Mo foil, GeS_2_-MoS_2_/rGO and MoS_2_/rGO. (h) WT contour plots at the Ge K-edge of Ge foil, GeS_2_-MoS_2_/rGO and GeS_2_/rGO. No Ge–Mo bond can be observed, suggesting that the heterointerfaces undergoes charge transfer mediated by S, which is consistent with the above DFT results.

To determine the valence and chemical coordination environment changes of the GeS_2_-MoS_2_ heterostructure, Mo and Ge K-edge X-ray absorption fine structure (XAFS) spectra were further measured. The X-ray absorption near-edge structure (XANES) spectra of the Mo K-edge in Mo foil, GeS_2_-MoS_2_/rGO and MoS_2_/rGO are displayed in Fig. 2(a). In the enlarged illustration, the pre-edge feature of GeS_2_-MoS_2_/rGO and MoS_2_/rGO show a shift to higher energy compared to that of Mo foil. Because Mo is oxidized to a higher state, the valence state of Mo in GeS_2_-MoS_2_/rGO is slightly higher than that in MoS_2_/rGO, which is consistent with the XPS results of Mo 3d (Fig. 1(b)). In addition, the Ge valence state in GeS_2_-MoS_2_/rGO is lower than that in GeS_2_/rGO (Fig. 2(b)), demonstrating the electron transfer from MoS_2_ to GeS_2_ in the heterointerfaces.^38^ According to the *R*-space of the extended X-ray absorption fine structure (EXAFS) in Fig. 2(c), the EXAFS spectra of GeS_2_-MoS_2_/rGO and MoS_2_/rGO exhibit two main peaks at around 1.9 and 2.6 Å, corresponding to Mo–S and Mo–Mo bonds, respectively.^39^ And the EXAFS spectra in Fig. 2(d) show that the GeS_2_-MoS_2_/rGO and GeS_2_/rGO peaks are similar to those of Ge foil, but the positions of the peaks are slightly lower than those of Ge foil, indicating that Ge is bonded with other elements. Thus, the EXAFS spectra of GeS_2_-MoS_2_/rGO and GeS_2_/rGO are fitted, corresponding to Ge–Ge and Ge–S bonds, respectively (Fig. S3†). Fig. 2(e and f) display the oscillation curves of the Mo and Ge K-edge for different samples in the 0–12 Å^−1^ K range. The decrease in the oscillation intensity suggests a periodic decrease, which is due to the formation of rich heterointerfaces between the two crystal phases (MoS_2_ and GeS_2_) in GeS_2_-MoS_2_/rGO by charge transfer. The wavelet transform (WT) is considered to be a good complement to the Fourier-transform (FT) for separating backscattered atoms in both *R*-space and *K*-space resolution and displaying atomic dispersion.^40^Fig. 2(g and h) exhibit the Mo K-edge and Ge K-edge WT results of different samples, respectively. For GeS_2_-MoS_2_/rGO, MoS_2_/rGO, Mo foil, GeS_2_/rGO, and Ge foil, the fitted parameters of the *R*-space are shown in Tables S2 and S3,† which further suggests that the lattice distortion and interfacial charge redistribution are expected to contribute to the electrochemical performance of LSBs. Furthermore, the absence of Ge–Mo bonds in the GeS_2_-MoS_2_ heterostructure indicates that the heterointerfaces are mediated by S for charge transfer, which is consistent with the DFT results.

### 2.2 Lattice-matching nature between GeS_2_ and Li_2_S

The material ratios are optimized based on the morphology and cycling capacity of the GeS_2_-MoS_2_/rGO heterostructure, and the element content (Table S4†) was measured by inductively coupled plasma-optical emission spectrometry (ICP-OES). Heterostructures with three different ratios (MoS_2_ : GeS_2_ = 0.7, 0.9 and 1.1) were prepared for morphology and capacity characterization (Fig. S4†). When MoS_2_ : GeS_2_ = 0.7, a large amount of GeS_2_ accumulates on the surface of MoS_2_. This hinders the contact between MoS_2_ and LiPSs and weakens the adsorption effect of the heterostructure on LiPSs, leading to a rapid capacity decay (capacity retention of 77.29% after 300 cycles at 0.5C). When MoS_2_ : GeS_2_ = 1.1, MoS_2_ agglomerates heavily in the heterostructure, and only a small number of GeS_2_ layers are attached to the MoS_2_ surface. The reduced pores lead to a decrease in contact between the electrolyte and the material, which affects ionic conduction and ultimately electrochemical performance (capacity retention of 79.51% after 300 cycles at 0.5C). When MoS_2_ : GeS_2_ = 0.9, the hierarchical heterostructure can significantly expand the contact area between the electrode and electrolyte, and therefore increase the active reaction and storage sites for LiPSs and Li_2_S. The nanosheets in the heterostructure can greatly shorten the ion transport path, which increases the reversible capacity of the battery (capacity retention of 89.17% after 300 cycles at 0.5C). As a result, in this work, we chose a heterostructure with a MoS_2_ to GeS_2_ ratio of 0.9 as the sulfur host for the study, obtaining excellent electrochemical cycling preformance and enhanced redox kinetics.

**Fig. 3:**
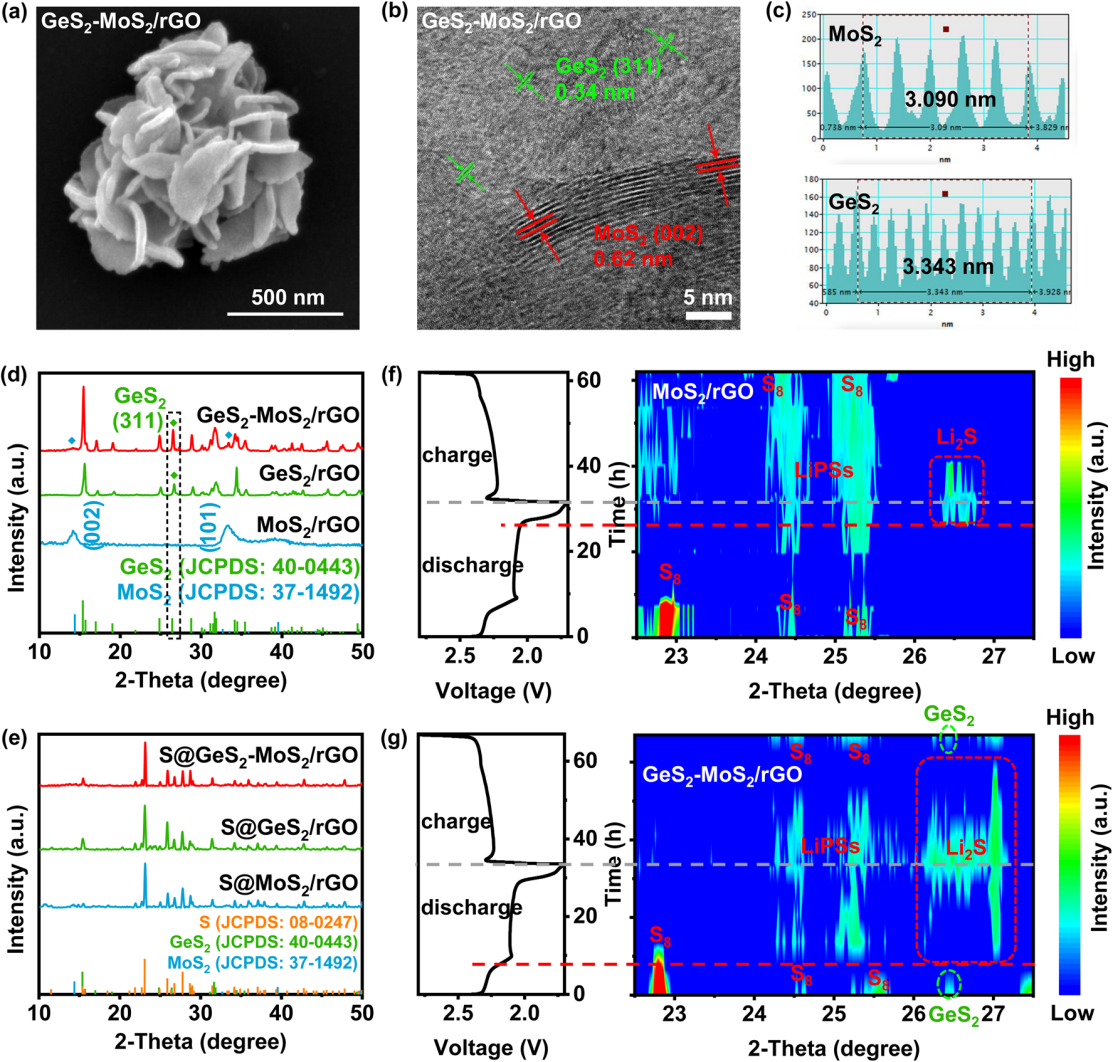
(a) SEM images of GeS_2_-MoS_2_/rGO. A petal-spherical morphology can be observed. (b) HRTEM image of GeS_2_-MoS_2_/rGO and (c) the corresponding reduced FFT patterns. The GeS_2_ (311) and MoS_2_ (002) lattice fringes can be determined. (d) XRD patterns of GeS_2_-MoS_2_/rGO, MoS_2_/rGO and GeS_2_/rGO. (e) XRD patterns of S@GeS_2_-MoS_2_/rGO, S@MoS_2_/rGO and S@GeS_2_/rGO. (f and g) The initial discharge/charge profiles and corresponding *in situ* XRD contour plots of MoS_2_/rGO and GeS_2_-MoS_2_/rGO batteries, respectively. The lattice-matching nature between *Fdd*2 GeS_2_ and *Fm*3̄*m* Li_2_S significantly advances the Li_2_S deposition at about 60% SOC for the GeS_2_-MoS_2_/rGO battery. Also, the GeS_2_-MoS_2_/rGO battery exhibits a much weaker and reversible LiPS peak compared with the MoS_2_/rGO battery, implying that the LiPS shuttling is significantly restrained by the GeS_2_-MoS_2_/rGO “butterfly material”.

The morphology of the prepared GeS_2_-MoS_2_/rGO was characterized, which confirmed the formation of the heterostructure and heterointerfaces. As shown by the scanning electron microscopy (SEM) and transmission electron microscopy (TEM) results (Fig. 3(a) and S5(a, b)),† the GeS_2_-MoS_2_/rGO heterostructure exhibits uniformly hierarchical petal-spherical particles. And the nanosheets in GeS_2_-MoS_2_/rGO clearly show hierarchical structures, indicating that the heterostructure effectively expands the contact area between the electrode and electrolyte as well as exposes abundant active sites. These advantages can significantly shorten the ion transport path and improve the kinetics of the reaction. The interface of the GeS_2_-MoS_2_/rGO heterostructure is shown in the high-resolution TEM (HRTEM) image (Fig. 3(b)). The lattice fringe spacing of 0.62 nm is assigned to the (002) plane of hexagonal MoS_2_, and 0.34 nm corresponds to the (311) plane of orthorhombic GeS_2_, further revealing the formation of the heterostructure. According to the energy dispersive X-ray (EDX) analysis (Fig. S5(c–f)),† the distribution of S, Mo, and Ge elements of GeS_2_-MoS_2_/rGO is clearly observed, where GeS_2_ nanosheets are dispersed on the MoS_2_ samples. Atomic force microscopy (AFM) measurement was carried out to determine the thickness of the nanosheets in GeS_2_-MoS_2_/rGO (Fig. S6†). The results show that the particle distribution of GeS_2_-MoS_2_/rGO is 100–500 nm with a thickness of about 6 nm. The ultra-thin nanosheets effectively shorten the ion and electron transport paths and accelerate the surface charge transfer rate, ultimately improving the redox kinetics of LSBs. In addition, the morphology analyses of MoS_2_/rGO and GeS_2_/rGO can also prove the successful synthesis of materials and the uniform distribution of elements (Fig. S7 and S8†). More interestingly, the EDX results of GeS_2_-MoS_2_/rGO show that GeS_2_ nanosheets are generated more on the outer petals in the hierarchical GeS_2_-MoS_2_/rGO, suggesting that GeS_2_ nanosheets in the heterostructure grow epitaxially along the conductive MoS_2_ core. These unique hierarchical petal-spherical GeS_2_-MoS_2_/rGO heterostructures provide an effectively shortened ion transport path, which facilitates redox kinetics of LSBs.

The X-ray diffraction (XRD) pattern of GeS_2_-MoS_2_/rGO in Fig. 3(d) displays the characteristic diffraction peaks of orthorhombic GeS_2_ (JCPDS no. 40-0443) and two diffraction peaks at 14.38° and 32.68° which correspond to hexagonal MoS_2_ (JCPDS no. 37-1492). The XRD result of GeS_2_-MoS_2_/rGO shows mixed peaks of GeS_2_ and MoS_2_ phases, implying the coexistence of GeS_2_ and MoS_2_ to construct the heterostructure. And the XRD patterns (Fig. 3(e)) of different catalysts after sulfur loading exhibit the presence of a cubic sulfur crystal structure (JCPDS no. 08-0247). In addition, the sulfur content was determined with a thermogravimetric (TG) analyzer (Fig. S9†), and the sulfur contents of GeS_2_-MoS_2_/rGO, GeS_2_/rGO and MoS_2_/rGO are approximately 71.7, 71.9 and 71.6 wt%, respectively. Fig. S10† exhibits the Brunauer–Emmett–Teller (BET) results of various catalysts. And the specific surface area (SSA) of the GeS_2_-MoS_2_/rGO heterostructure is 141.232 m^2^ g^−1^, which is well above that of MoS_2_/rGO (57.085 m^2^ g^−1^) and GeS_2_/rGO (2.434 m^2^ g^−1^). This result further demonstrates that the hierarchical heterostructure exposes more active sites and increases the contact area between the electrode and electrolyte.

As discussed previously, the lattice-matching nature between *Fdd*2 GeS_2_ and *Fm*3̄*m* Li_2_S can induce Li_2_S multi-site nucleation and 3D growth. The evolution of sulfur species during the electrochemical process was monitored by *in situ* characterization (Fig. 3(f and g)). Throughout the electrochemical reaction, *in situ* XRD shows the conversion from S_8_ to LiPSs and finally to Li_2_S. For the MoS_2_/rGO battery (Fig. 3(f)), at the beginning of the discharge process, the XRD diffraction peaks of S_8_ can be clearly seen. The broad peak at 24–25.5° corresponds to long-chain LiPSs.^41,42^ The Li_2_S peak appeared at about 20% SOC, and corresponds to the posterior liquid-to-solid or solid-to-solid discharge intervals for LSBs. Also, we can find that the LiPS peak for the MoS_2_/rGO battery is broad and strong, and corresponds to more accumulated liquid LiPSs and a severe shuttle effect. However, in comparison, for the GeS_2_-MoS_2_/rGO battery (Fig. 3(g)), a characteristic peak of cubic Li_2_S (111) appears at 26.3–27° at high SOC (80% SOC), which is superior to that of the MoS_2_/rGO battery (60% SOC). The Li_2_S peak is in accordance with the orthorhombic GeS_2_ (311) plane (26.4°),^43^ suggesting that the lattice-matching nature between *Fdd*2 GeS_2_ and *Fm*3̄*m* Li_2_S significantly advances the Li_2_S deposition. Also, we can observe that the LiPS peak for the GeS_2_-MoS_2_/rGO battery was much weaker, and it disappeared after the battery was fully charged. By comparing these results with the Li_2_S peak at 20% SOC which appeared late and the LiPS peak at 100% SOC which did not disappear, of the MoS_2_/rGO battery, we can claim that the accumulation of LiPSs is significantly restrained in the GeS_2_-MoS_2_/rGO battery.

### 2.3 Synergistic “butterfly” to realize Li_2_S 3D growth

**Fig. 4:**
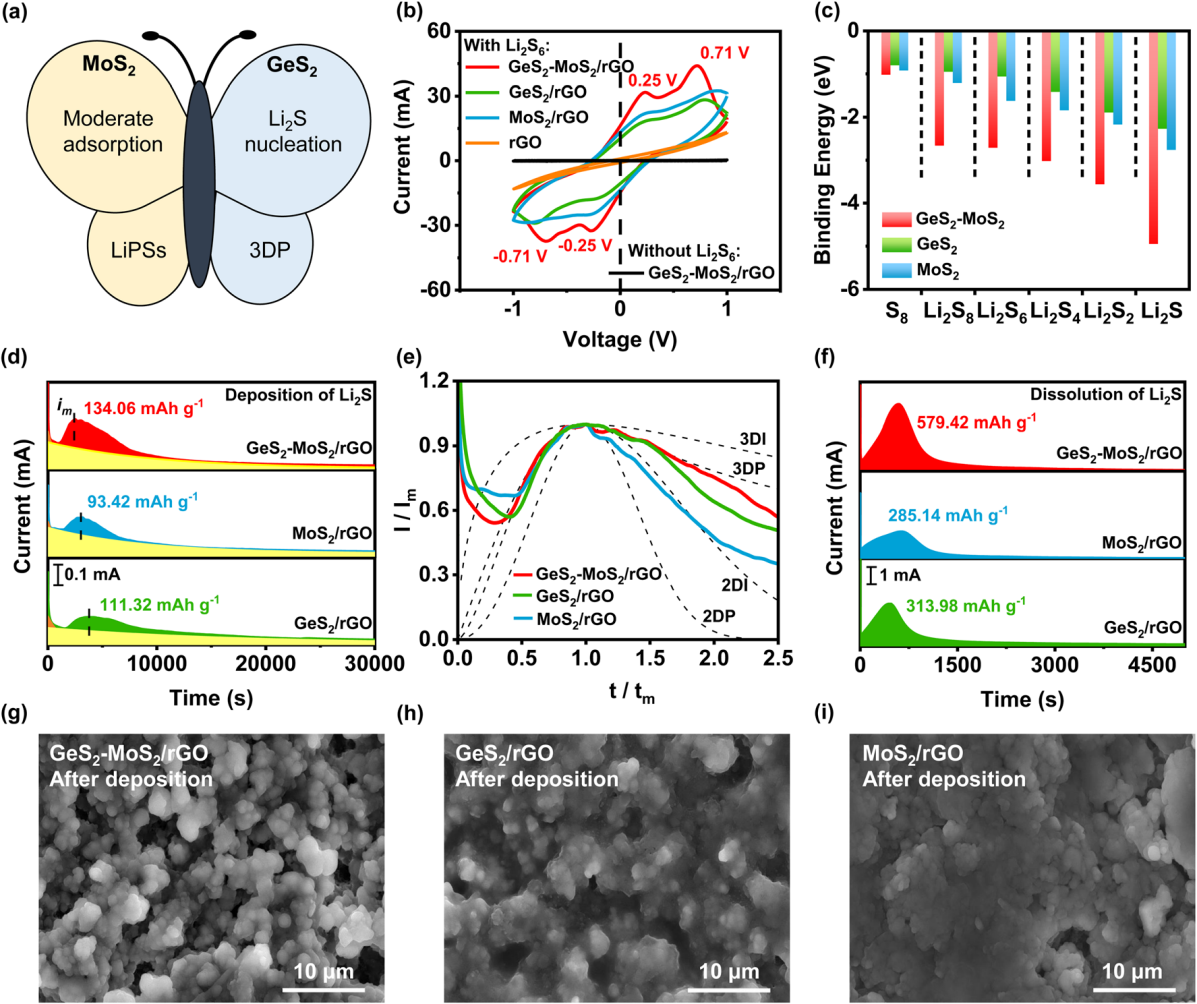
(a) Schematic illustration of the GeS_2_-MoS_2_ heterostructure. The butterfly heterostructure is designed as a sulfur host to facilitate the conversion of LiPSs and promote the growth of Li_2_S synergistically. (b) CV curves of different catalysts in symmetric batteries at a scan rate of 20 mV s^−1^. GeS_2_-MoS_2_/rGO has the strongest peak current density and the smallest overpotential; (c) binding energies of S_8_ and Li_2_S_*x*_ (*x* = 1, 2, 4, 6, and 8) species on different catalysts. The GeS_2_-MoS_2_ heterostructure has the strongest binding energy with LiPSs, effectively promoting the catalytic effect. (d) Potentiostatic discharge profiles at 2.05 V with Li_2_S_8_ catholyte on different samples. The GeS_2_-MoS_2_/rGO heterostructure with rich catalytic heterointerfaces can achieve rapid conversion of LiPSs and advanced deposition of Li_2_S. (e) Dimensionless transient (symbols) of different samples in comparison with theoretical 2D and 3D models (*I*_m_: peak current; *t*_m_: time needed to achieve the peak current). Li_2_S growth follows a typical 3DP mode in the GeS_2_-MoS_2_/rGO heterostructure. (f) Potentiostatic charge profiles of different samples at 2.40 V for Li_2_S dissolution. (g–i) SEM images of GeS_2_-MoS_2_/rGO, GeS_2_/rGO and MoS_2_/rGO after Li_2_S deposition. The uniform and radial deposition of Li_2_S is achieved on the surface of the GeS_2_-MoS_2_/rGO heterostructure.

The GeS_2_-MoS_2_ heterostructure can effectively catalyze liquid–solid reaction kinetics and regulate the Li_2_S growth process (Fig. 4(a)). To investigate the electrochemical stability and electrocatalytic activity of different catalysts toward polysulfide conversion, cyclic voltammetry (CV) tests were performed in symmetric batteries at a scan rate of 20 mV s^−1^ (Fig. 4(b)). GeS_2_-MoS_2_/rGO exhibits two reduction peaks at −0.25 and −0.71 V, which are related to the reduction of S_8_ to Li_2_S_6_ and then Li_2_S_6_ to Li_2_S_2_/Li_2_S. And the latter well-defined oxidation peaks at 0.25 and 0.71 V are associated with the oxidation of Li_2_S_2_/Li_2_S to Li_2_S_6_ and Li_2_S_6_ to S_8_.^44,45^ The CV curve of GeS_2_-MoS_2_/rGO without Li_2_S_6_ electrolyte presents a characteristic rectangle of pure capacitive behavior, indicating that Li_2_S_6_ is the only electrochemically active species. Moreover, the stronger peak current densities and smaller overpotential (Δ*E*) of GeS_2_-MoS_2_/rGO compared to GeS_2_/rGO and MoS_2_/rGO indicate more robust interfacial stability and electrocatalytic performance.^46^

The interactions between different catalysts and LiPSs were investigated by calculating the adsorption energies of various hosts on LiPSs (Fig. 4(c)). And the optimized adsorption configurations of LiPS (Li_2_S, Li_2_S_2_, Li_2_S_4_, Li_2_S_6_, Li_2_S_8_, and S_8_) species on the GeS_2_-MoS_2_ heterostructure, and MoS_2_ (002) and GeS_2_ (311) surfaces are displayed in Fig. S11–S13.†Fig. 4(c) shows the strongest binding energies (*E*_b_) between the GeS_2_-MoS_2_ heterostructure and LiPSs, which indicates that their strong interactions can effectively balance the adsorption/dissociation and catalytic performance. Overall, the DFT results demonstrate that the heterostructure after the addition of GeS_2_ to MoS_2_ enhances the chemisorption ability of LiPSs with strong charge transfer and multiple adsorption sites.

To reveal the LiPS trapping ability of the as-prepared catalysts, the adsorption experiments were conducted by immersing the samples with the same content in Li_2_S_6_ solution (Fig. S14†). In the adsorption optical images in Fig. S14(a),† the original orange-brown solution containing GeS_2_-MoS_2_/rGO becomes slightly lighter after 1 h compared to the other two hosts, and all catalysts become colorless after 6 h. Furthermore, *ex situ* ultraviolet-visible (UV-vis) absorption spectra were examined to evaluate the concentration changes of the Li_2_S_6_ solution used. As shown in Fig. S14(b),† the Li_2_S_6_ absorption band in the 400–500 nm region almost disappears for the GeS_2_-MoS_2_/rGO host, which demonstrates that GeS_2_-MoS_2_/rGO can effectively anchor LiPSs. These results suggest a strong chemical interaction between GeS_2_-MoS_2_/rGO and LiPSs, which is attributed to the rich interfacial interaction of the heterostructure.

Li_2_S nucleation and dissolution experiments were conducted to study the liquid–solid reaction kinetics and Li_2_S deposition process of different catalysts.^47^ According to Faraday's law, the Li_2_S deposition capacities of GeS_2_-MoS_2_/rGO, GeS_2_/rGO, and MoS_2_/rGO were calculated to be 134.06, 111.32, and 93.42 mA h g^−1^, respectively (Fig. 4(d)). The growth of Li_2_S is closely related to the deposition kinetics, which determines the deposition capacity of Li_2_S and the reversibility of LSBs. Cui *et al.*^48^ preliminarily demonstrated that the polar sites can strongly adsorb LiPSs and significantly reduce the interfacial impedance of Li_2_S deposition. First, Li_2_S nucleates on the cathode substrate by overcoming the interfacial impedance between the electrolyte and the substrate. Subsequently, LiPSs will be converted to Li_2_S by adsorption and simultaneously precipitated as Li_2_S.^49^ As shown in Fig. 4(d), the depositional curve shows a clear hill-like shape, with a period of incubation ahead (when the current reaches *i*_m_). And the incubation process is related to the reduction of long chain LiPSs (*i.e.*, Li_2_S_8_ and Li_2_S_6_) to short chain Li_2_S_4_. Because of the better electrical conductivity of MoS_2_ than GeS_2_, MoS_2_ has a stronger adsorption effect on LiPSs, which can promote the conversion of LiPSs to Li_2_S (current reaches *i*_m_ faster). Compared with GeS_2_/rGO, the peak current of the Li_2_S deposition curve of MoS_2_/rGO appears earlier and the peak current is enhanced (peak current of MoS_2_/rGO is 0.19 mA at 3035 s and that of GeS_2_/rGO is 0.14 mA at 3747 s), indicating a faster response to Li_2_S nucleation. Moreover, the Li_2_S deposition process ends prematurely with MoS_2_/rGO, which is due to the lack of 3D nucleation leading to premature passivation of the cathode substrate. So, because of the lattice-matching nature between *Fdd*2 GeS_2_ and *Fm*3̄*m* Li_2_S, GeS_2_ can induce multi-site nucleation and 3D deposition of Li_2_S, and the deposition capacity of GeS_2_/rGO is higher than that of MoS_2_/rGO. More importantly, due to the synergistic effect of MoS_2_ and GeS_2_, the GeS_2_-MoS_2_/rGO heterostructure with rich catalytic heterointerfaces can achieve rapid conversion of LiPSs and high Li_2_S precipitation. Thus, the GeS_2_-MoS_2_/rGO heterostructure has the earliest Li_2_S deposition current (0.23 mA at 2518 s) and the highest deposition capacity. These results suggest that hierarchical GeS_2_-MoS_2_/rGO with rich catalytic heterointerfaces provides more active sites for achieving rapid conversion of LiPSs and advanced deposition of Li_2_S.

To investigate the Li_2_S growth behavior of different catalysts, a dimensionless diagnostic analysis of the current–time curves obtained from Li_2_S nucleation tests was conducted according to the Scharifker–Hills model (Fig. 4(e) and eqn (S1)–(S4)†).^42,50^ Four classical electrochemical deposition models are used to fit the current–time responses obtained in chronoamperometric tests. Among them, two-dimensional progressive (2DP) and two-dimensional transient (2DI) nucleation are controlled by incorporating adatoms into the lattice interface. And three-dimensional progressive (3DP) and three-dimensional transient (3DI) nucleation are achieved by volume diffusion controlled growth.^51^ For GeS_2_/rGO, Li_2_S growth shows a mixed 2DI and 3DP mode, while a typical 2DI mode is presented in MoS_2_/rGO. In comparison, because of the lattice-matching nature between *Fdd*2 GeS_2_ and *Fm*3̄*m* Li_2_S, GeS_2_ induces the deposition and growth of Li_2_S during the discharge process, thus showing a tendency of 3D model. However, the typical 3DP model is not presented in GeS_2_/rGO, probably due to the weak electrical conductivity and insufficient reactive sites of GeS_2_. In the GeS_2_-MoS_2_/rGO heterostructure, Li_2_S growth follows a typical 3DP mode. GeS_2_ is grown on the epitaxial petals of conductive-core MoS_2_, and lattice-matching between *Fdd*2 GeS_2_ and *Fm*3̄*m* Li_2_S induces rapid and multi-site nucleation of Li_2_S on the surface of the heterostructure, which enables the multi-site deposition and 3D growth of Li_2_S.^25,52^

Furthermore, Li_2_S dissolution experiments verified the excellent kinetic properties of GeS_2_-MoS_2_/rGO (Fig. 4(f)). As a result, the GeS_2_-MoS_2_/rGO catalytic electrode exhibits higher current density and Li_2_S dissolution capacity (579.42 mA h g^−1^) compared to the GeS_2_/rGO (285.14 mA h g^−1^) and MoS_2_/rGO (313.98 mA h g^−1^) electrodes. The first step of solid–solid decomposition of Li_2_S is the slowest step in the charge process, resulting in ultra-high overpotential. These results indicate that the introduced GeS_2_-MoS_2_/rGO catalyst can effectively reduce the decomposition barriers and accelerate the charge process.

The phenomenon of Li_2_S growth can also be obtained from the SEM morphologies of the deposited electrodes (Fig. 4(g–i)). Fig. 4(g) shows the uniform and radial deposition of Li_2_S on the GeS_2_-MoS_2_/rGO heterostructure. And the deposition of Li_2_S on GeS_2_/rGO has a tendency of radial growth, which is in agreement with the deposition model and lattice-matching (Fig. 4(e)). However, the deposition of Li_2_S on MoS_2_/rGO forms a dense coating covering the catalyst, which hindered the subsequent Li_2_S deposition (Fig. 4(i)). The 3DP model of Li_2_S nucleation in GeS_2_-MoS_2_/rGO suggests that the sufficient active sites of heterointerfaces and the lattice-matching between *Fdd*2 GeS_2_ and *Fm*3̄*m* Li_2_S can guide the radial Li_2_S growth, thus balancing surface transverse atomic diffusion and mass transport in the electrolyte.^53^ Therefore, the large accumulation of Li_2_S caused by the passivation of the electrode surface can be effectively avoided, as demonstrated in Fig. 1(d). Li_2_S 3D growth of GeS_2_-MoS_2_/rGO shortens the ion/electron diffusion path and exposes sufficient catalytically active sites for Li_2_S conversion. More importantly, the ionic and electronic conduction networks are always present on the surface of the heterostructure, thus consistently providing an efficient pathway for LiPS conversion as well as excellent redox kinetics.

### 2.4 Electrochemical performance of LSBs

**Fig. 5:**
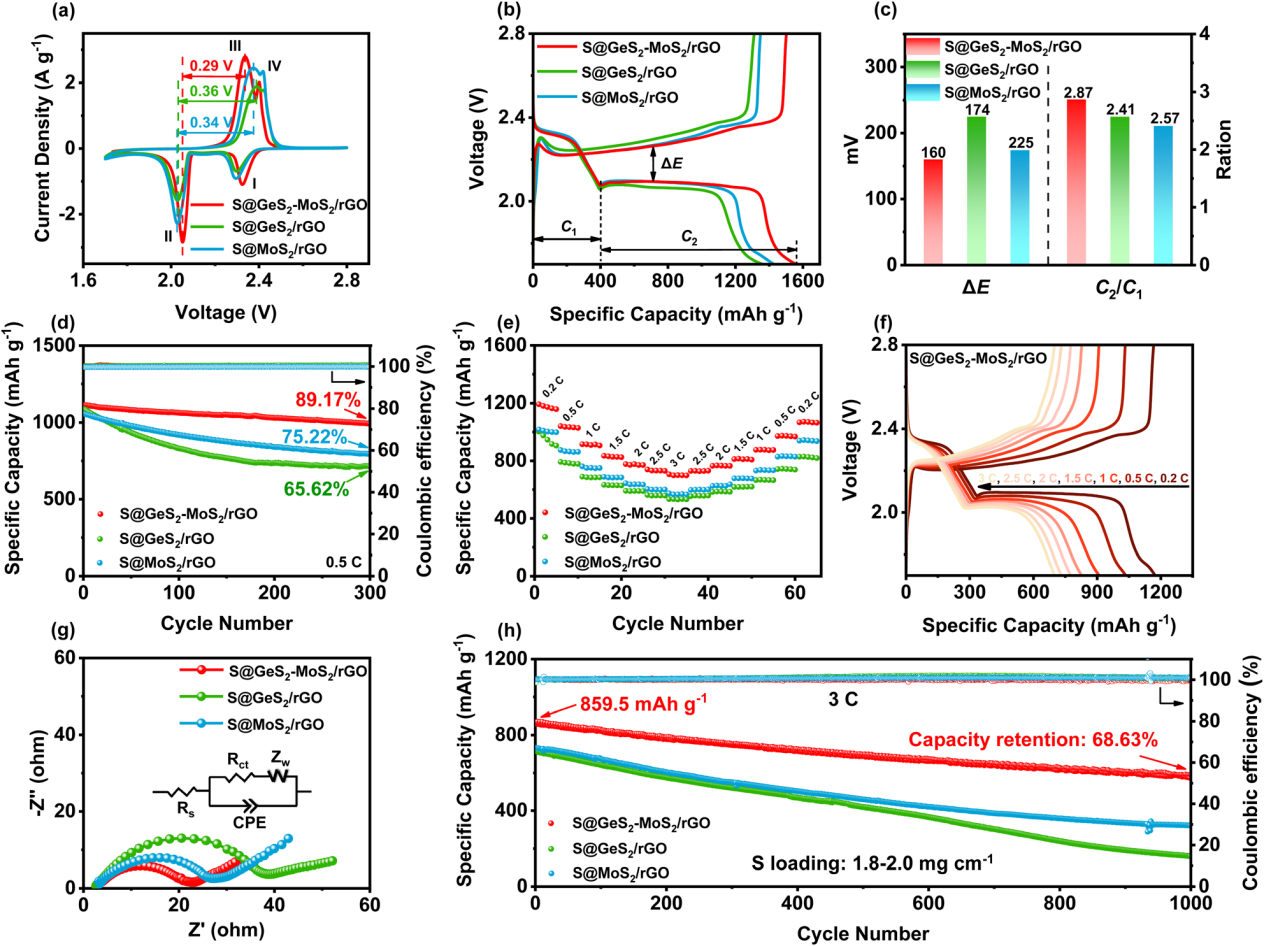
(a) CV curves of different electrodes at a scan rate of 0.1 mV s^−1^ within 1.7–2.8 V. The S@GeS_2_-MoS_2_/rGO cathode shows the lowest potential, indicating enhanced redox kinetics. (b) Galvanostatic discharge/charge profiles of different electrodes at a 0.1C current rate. (c) Δ*E* and *C*_2_/*C*_1_ values of galvanostatic discharge/charge profiles. The S@GeS_2_-MoS_2_/rGO cathode exhibits the lowest polarization potential and highest *C*_2_/*C*_1_ value, demonstrating its excellent electrocatalytic activity. (d) Cycling life of different electrodes at 0.5C over 300 cycles. (e) Rate performance of different electrodes with various current densities. The S@GeS_2_-MoS_2_/rGO electrode displays the highest capacity retention and rate capacities. (f) Galvanostatic discharge/charge profiles of S@GeS_2_-MoS_2_/rGO at various current densities. (g) EIS spectra of different cathodes after 100 cycles. GeS_2_-MoS_2_/rGO exhibits a minimal *R*_ct_ value, which is attributed to the excellent electrical conductivity of the heterostructure. (h) Cycling stability of different electrodes at 3C over 1000 cycles. The S@GeS_2_-MoS_2_/rGO electrode shows a high discharge capacity and stable cycling performance, indicating that LiPS shuttling during cycling is effectively suppressed.

CV tests were performed on S@GeS_2_-MoS_2_/rGO, S@MoS_2_/rGO and S@GeS_2_/rGO cathodes to investigate the redox kinetics of different catalysts (Fig. 5(a)). All cathodes show two representative cathodic and anodic peaks, respectively. The two cathodic peaks (peak I and peak II) are attributed to the reduction of S_8_ molecules to long-chain LiPSs (Li_2_S_*x*_, 4 ≤ *x* ≤ 8; peak I) and their subsequent reduction to short-chain sulfides (peak II). The anodic peaks (peak III and peak IV) originate from the oxidation of short-chain sulfides eventually to S_8_.^54^ As shown in Fig. 5(a), the reduction peak of S@GeS_2_-MoS_2_/rGO at about 2.0 V shifts significantly to a higher potential compared with that of S@GeS_2_/rGO and S@MoS_2_/rGO, demonstrating a promoted conversion from LiPSs to Li_2_S. And the S@GeS_2_-MoS_2_/rGO cathode also shows the lowest oxidation potential in the oxidation process, indicating an enhanced Li_2_S oxidation reaction. These results demonstrate that the GeS_2_-MoS_2_/rGO heterostructure shows fast redox kinetics and high reversibility with the help of rich heterointerfaces and the synergistic effect.

The hierarchical nanosheets and strong catalytic heterointerfaces endow the S@GeS_2_-MoS_2_/rGO cathode with reduced polarization and stable cycling performance. In Fig. 5(b), the discharge/charge curves of different cathodes at 0.1C (1C = 1675 mA g^−1^) are compared, and the discharge and charge plateaus are consistent with the CV analysis. The voltage gap between the second discharge and the charge plateaus is the polarization potential (Δ*E*), denoting a hysteresis in the redox reaction, and the value of Δ*E* is taken at 50% of the discharge capacity. The S@GeS_2_-MoS_2_/rGO cathode exhibits a lower polarization potential (Δ*E* = 160 mV) than S@GeS_2_/rGO (Δ*E* = 225 mV) and S@MoS_2_/rGO cathodes (Δ*E* = 174 mV), due to the excellent electrocatalytic activity of GeS_2_-MoS_2_/rGO for LiPS conversion.

*C*_1_ and *C*_2_ are defined as the capacities of the two discharge plateaus, respectively (Fig. 5(c)). And the ratio *C*_2_/*C*_1_ can be explained by the catalytic activity of the LiPS conversion reaction. *C*_1_ represents the amount of liquid LiPSs produced (S_8_ → S_6_^2−^ → S_4_^2−^), and *C*_2_ represents the efficiency of reducing LiPSs to Li_2_S (S_4_^2−^ → Li_2_S_2_ → Li_2_S).^55^ Therefore, the higher the *C*_2_/*C*_1_, the better the catalytic ability. The slow kinetics and the shuttle effect caused by the diffusion of liquid LiPSs in the discharge process lead to a decrease in the capacity between the *C*_1_ and *C*_2_ stages.^56^ As shown in Fig. 5(c), the *C*_2_/*C*_1_ of S@GeS_2_-MoS_2_/rGO is 2.87, much higher than that of S@GeS_2_/rGO (2.41) and S@MoS_2_/rGO (2.57), which further confirmed the superior catalytic activity of the GeS_2_-MoS_2_/rGO heterostructure toward the LiPS redox reaction.

The cycling performance of different electrodes is tested at a current of 0.2C (Fig. S15†). Among the three electrodes, the S@GeS_2_-MoS_2_/rGO cathode shows the highest capacity and the best cycling stability, with a high capacity retention of 90.10% after 300 cycles. In contrast, the S@GeS_2_/rGO and S@MoS_2_/rGO electrodes deliver a lower capacity retention of 79.16% and 84.85%, respectively. The excellent electrochemical performance of the battery with the S@GeS_2_-MoS_2_/rGO electrode is mainly attributed to the improved electronic conductivity and rich catalytic heterointerfaces of the GeS_2_-MoS_2_/rGO heterostructure.

The cycling performance of various cathodes at 0.5C is displayed in Fig. 5(d). Among the three cathodes, the S@GeS_2_-MoS_2_/rGO electrode delivers the highest initial capacity of 1114.5 mA h g^−1^ at 0.5C and stabilizes at 993.8 mA h g^−1^ over 300 cycles. The S@GeS_2_-MoS_2_/rGO electrode also maintains the highest capacity retention at 89.17%, indicating excellent reaction kinetics and cycling stability. On the other hand, the S@GeS_2_/rGO and S@MoS_2_/rGO electrodes show discharge capacities of 714.0 and 794.8 mA h g^−1^ after 500 cycles with a capacity retention of 65.62% and 75.22%, respectively. The lower capacity retention of these two cathodes is mainly related to the rapid dissolution of LiPSs into the electrolyte. These results demonstrate that the S@GeS_2_-MoS_2_/rGO electrode achieves limited LiPS shuttling as well as fast sulfur reaction kinetics because of the rich catalytic heterointerfaces and advanced deposition of Li_2_S in the GeS_2_-GeS_2_-MoS_2_/rGO heterostructure.

The rate performance of the three cathodes at various current densities in the range of 0.2 to 3C is presented in Fig. 5(e). Clearly, LSBs with the GeS_2_-MoS_2_/rGO catalyst deliver the highest rate performance in different cathodes. The discharge capacities of S@GeS_2_-MoS_2_/rGO are 1173.3, 1034.6, 909.5, 827.8, 776.7, 732.2 and 700.2 mA h g^−1^ at 0.2, 0.5, 1, 1.5, 2, 2.5 and 3C, respectively, while the batteries using S@GeS_2_/rGO and S@MoS_2_/rGO cathodes show lower capacities. At a current density of 3C, the capacity retention of the S@GeS_2_-MoS_2_/rGO electrode is 59.7%, much higher than that of S@GeS_2_/rGO (56.2%) and S@MoS_2_/rGO (56.5%) electrodes, indicating a significantly higher sulfur utilization and improved LiPS conversion of the GeS_2_-MoS_2_/rGO heterostructure. Fig. 5(f) displays the discharge/charge profiles of the S@GeS_2_-MoS_2_/rGO electrode. The potential gap between the discharge and charge plateaus gradually increases with increasing current density. However, even at high current densities of 3C, two distinct discharge plateaus can still be obtained, which indicates the fast reaction kinetics of LiPSs in the GeS_2_-MoS_2_/rGO catalyst. Meanwhile, as shown in Fig. S16,† the corresponding discharge/charge voltage profiles of S@GeS_2_/rGO and S@MoS_2_/rGO cathodes show a larger polarization compared to S@GeS_2_-MoS_2_/rGO.

Electrochemical impedance spectroscopy (EIS) after 100 cycles further demonstrated the improved redox reactions of the GeS_2_-GeS_2_-MoS_2_/rGO heterostructure (Fig. 5(g)). In the equivalent circuit, the spot intersecting the horizontal axis is the interphase-contact resistance (*R*_s_) between the electrolyte and the battery. And the semicircle diameter at low frequencies indicates the charge-transfer resistance (*R*_ct_), which is related to the charge transfer between the electrode and the electrolyte on the electrode surface.^57^ According to the fitting results (Table S5†), the S@GeS_2_-MoS_2_/rGO electrode (16.52 Ω) has a smaller *R*_ct_ compared to the S@GeS_2_/rGO (33.64 Ω) and S@MoS_2_/rGO (24.37 Ω) electrodes. The battery using GeS_2_-GeS_2_-MoS_2_/rGO exhibits minimal *R*_ct_. This is attributed to the excellent electrical conductivity of the heterostructure, and the uniform precipitation and effective dissolution of Li_2_S, which is highly exposed to the catalytic surface after cycling.

Ultra-long cycling capabilities were tested at a high current density of 3C to explore the cycling stability of different catalysts (Fig. 5(h)). After 1000 cycles, the discharge capacity of the S@GeS_2_-MoS_2_/rGO electrode can be maintained as high as 589.9 mA h g^−1^, while those with S@GeS_2_/rGO and S@MoS_2_/rGO suffered a rapid capacity decay with retained capacities of 158.9 and 321.5 mA h g^−1^, respectively. The cycling capacity of different heterostructures at different current rates is compared in Table S6.† Most of the cathodes have excellent capacity retention at low current rates. Sulfur can be more easily embedded in the electrode material and form more stable chemical bonds at low current rates, resulting in better capacity retention. The capacity of LSBs decays more significantly at high rates. The dissolution and precipitation rate of electrode materials increase during cycling at high rates, and the migration rate of lithium ions in the electrolyte accelerates, which leads to a lower battery capacity retention. In this work, the S@GeS_2_-MoS_2_/rGO electrode delivers a high specific capacity and stable cycling performance, with a capacity retention of 68.63% and coulombic efficiency over 99.6% after 1000 cycles. The high cycling stability of the GeS_2_-MoS_2_/rGO battery suggests that the LiPS shuttling is effectively inhibited during electrochemical processes, which is attributed to the rich catalytic heterointerfaces and advanced deposition of Li_2_S in the heterostructure.

### 2.5 Low E/S ratio and LSB pouch battery performances

**Fig. 6:**
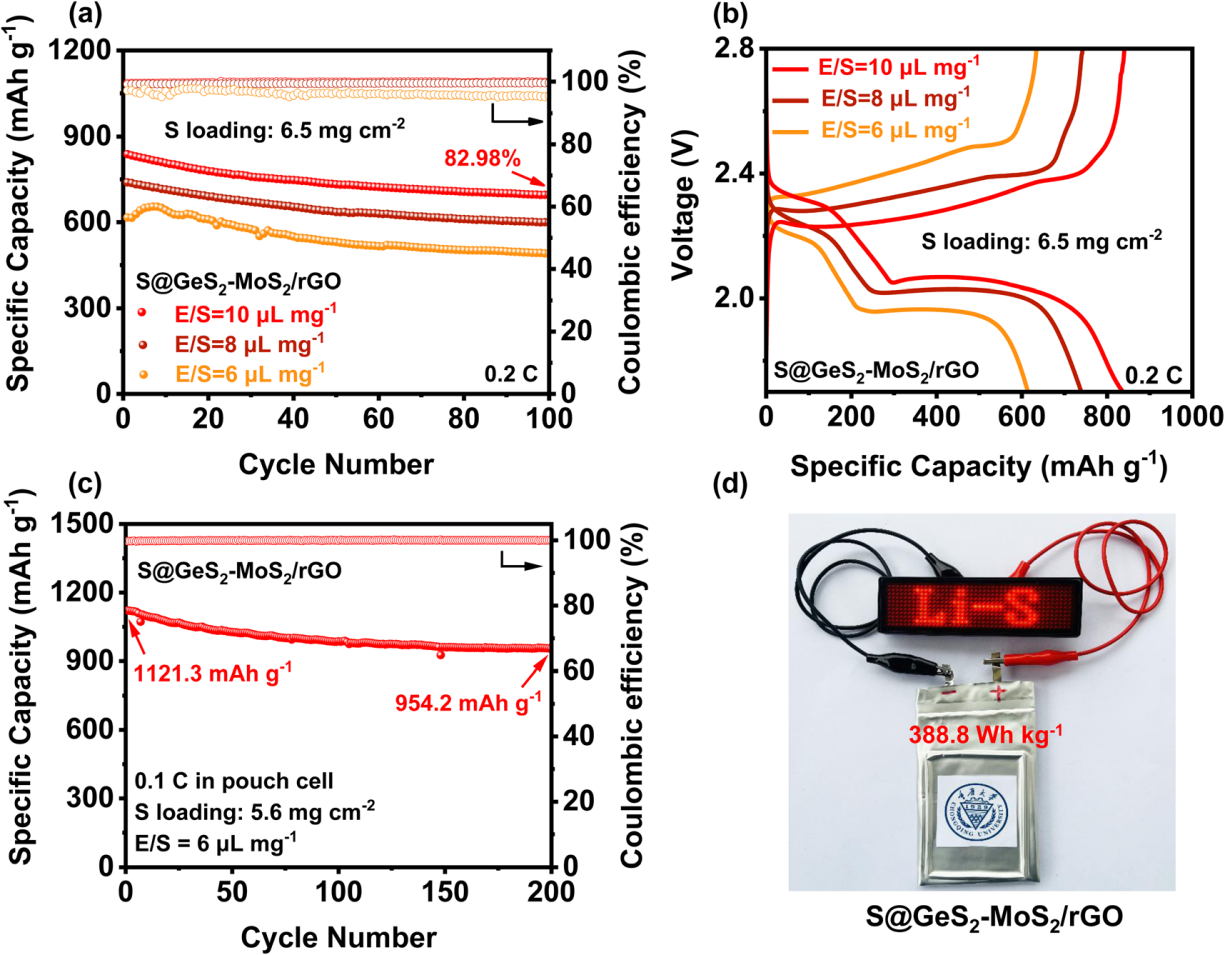
(a) Cycling performances of the S@GeS_2_-MoS_2_/rGO cathode with different E/S ratios at 0.2C. The S@GeS_2_-MoS_2_/rGO cathode obtains stable cycling and high capacity retention after 100 cycles, showing excellent sulfur utilization at low electrolyte usage. (b) Galvanostatic discharge/charge profiles of the S@GeS_2_-MoS_2_/rGO electrode with different E/S ratios at 0.2C. (c) Cycling stability of a pouch cell with the S@GeS_2_-MoS_2_/rGO electrodes at 0.1C. The S@GeS_2_-MoS_2_/rGO cathode maintains a high and stable capacity, suggesting potential electrochemical performance in practical applications. (d) Optical photograph of a pouch cell based on S@GeS_2_-MoS_2_/rGO electrodes charging a “Li–S” shaped LED.

To evaluate the practical applications for commercial LSBs, the electrochemical performances of the S@GeS_2_-MoS_2_/rGO electrode under high sulfur loading and in lean electrolyte were explored (Fig. 6(a and b)). With a sulfur loading of 6.5 mg cm^−2^ and E/S = 10 μL mg^−1^, the S@GeS_2_-MoS_2_/rGO cathode shows an initial discharge capacity of 837.9 mA h g^−1^ at 0.2C and a high capacity retention of 82.98% after 100 cycles. The cycling result of S@GeS_2_-MoS_2_/rGO displays a lower specific capacity when the *E*/*S* ratio decreases to 8 μL mg^−1^, but it still maintains a stable cycling performance. However, when the E/S ratio decreases to 6 μL mg^−1^, the S@GeS_2_-MoS_2_/rGO cathode exhibits an increasing trend in the first few cycles due to insufficient wetting of the electrode surface. This is attributed to the gradual infiltration and activation of the low amount of electrolyte in the highly loaded sulfur-active material. Even so, the S@GeS_2_-MoS_2_/rGO cathode still maintains a high stable cycling performance after 100 cycles, with a capacity retention rate of 79.57%. These results suggest that the GeS_2_-MoS_2_/rGO catalyst shows superiority in achieving good sulfur electrochemistry with low electrolyte usage.

To further approach the practical applicability of LSBs, pouch LSB cells with the S@GeS_2_-MoS_2_/rGO cathode were also fabricated and investigated (Fig. 6(c, d) and S17†). The result in Fig. 6(c) reveals a stable cycling stability at 0.1C, achieving a high initial capacity of 1121.3 mA h g^−1^ and an excellent discharge capacity of 954.2 mA h g^−1^ after 200 cycles. More importantly, the pouch cell can attain a practical specific energy of 388.8 W h kg^−1^. Even after 200 cycles, the energy density is still over 330 Wh kg^−1^. Besides, the charged light-emitting diode (LED) can be easily lit (Fig. 6(d)). The excellent electrochemical performance of the pouch battery can be attributed to the outstanding synergetic effect and rich heterointerfaces of the GeS_2_-MoS_2_/rGO heterostructure. All these results suggest that the GeS_2_-MoS_2_/rGO heterostructure has the potential to help LSBs reach practical applications.

## 3 Conclusion

The transformation of S_8_ into Li_2_S is found to be cross-executed rather than stepwise under practical working conditions for LSBs. Advancing the Li_2_S deposition can reduce the accumulation of liquid polysulfides and therefore increase the stability of the LSBs. Therefore, we designed a hierarchical petal-spherical GeS_2_-MoS_2_ “butterfly” material to accelerate the conversion of LiPSs and the deposition of Li_2_S simultaneously. The rich catalytic heterointerfaces and the lattice-matching nature between *Fdd*2 GeS_2_ and *Fm*3̄*m* Li_2_S enhance the adsorption of LiPSs and guide the Li_2_S growth in a 3D model, thus always providing transport channels for electrons and ions and improving the redox reaction kinetics. The above advantages realized the advanced deposition of Li_2_S from 20% to about 80% SOC in the discharge process, thereby achieving robust LSBs. The designed cathodes show excellent long-term cycling performance with a capacity retention of 68.63% at 3C over 1000 cycles. A high initial capacity of 837.9 mA h g^−1^ is achieved at a high sulfur loading of 6.5 mg cm^−2^ and a low E/S ratio of 10 μL mg^−1^. Moreover, a pouch LSB battery using S@GeS_2_-MoS_2_/rGO electrodes can attain a practical specific energy of 388.8 W h kg^−1^. Based on these fascinating advantages, this work provides a useful avenue for designing heterostructural catalysts for batteries and other advanced energy storage.

## 4 Methods

### 4.1 Materials

First, 2.0 g of GeO_2_ powder was heated in a mixed atmosphere of H_2_ and N_2_ at a volume ratio of 1 : 8 for 4 h at 700 °C to produce the precursor Ge powder. And graphene oxide (GO) was synthesized from natural graphite by the modified Hummers' method.^58^ Second, 0.1 g GO and 1.0 g Ge powder were evenly ground, dispersed in 20.0 mL of deionized water (DI), and stirred in a water bath at 50 °C to form a homogeneous solution. The obtained solution was freeze-dried for 24 h in a vacuum at −50 °C to obtain the dried Ge/GO composite. Finally, GeS_2_ mixed with reduced graphite oxide (rGO) samples (GeS_2_/rGO) was obtained by adding sulfur powder to Ge/rGO composites (molar ratio of 2 : 1) and calcining at 500 °C for 4 h under N_2_ conditions. Typically, 1.5 g (NH_4_)_6_Mo_7_O_24_·4H_2_O and 3.0 g CH_3_CSNH_2_ were dissolved in 100 mL DI and then 0.6 g polyvinyl pyrrolidone (PVP) was added. After the solution was mixed evenly, 0.05 g GO was added and stirred in a water bath at 50 °C for 4 h. After that, the solution was transferred to an oven and heated at 180 °C for 24 h. The black precipitate was collected by washing with DI and freeze-drying for 24 h. At last, MoS_2_/rGO composites were obtained by annealing in a N_2_ atmosphere at 500 °C for 4 h.

The synthesis of GeS_2_-MoS_2_/rGO is similar to that of MoS_2_/rGO. First, 1.5 g (NH_4_)_6_Mo_7_O_24_·4H_2_O, 3.0 g CH_3_CSNH_2_ and 0.6 g PVP were added to 100 mL of DI. Then 0.05 g GO and 0.5 g Ge were added to the mixed solution and stirred in a water bath at 50 °C for 4 h. Next, the mixture was heated in an oven at 180 °C for 24 h and then freeze-dried to obtain a dry black powder. Finally, an appropriate amount of sulfur powder was added to the black powder and calcined at 500 °C for 4 h to obtain GeS_2_-MoS_2_/rGO samples.

### 4.2 Electrode preparation

Three different cathodes were synthesized by mixing sulfur powder and the prepared samples (GeS_2_/rGO, MoS_2_/rGO and GeS_2_-MoS_2_/rGO) in a mass ratio of 7 : 3. The mixture was heated to 155 °C under a flowing N_2_ atmosphere for 12 h. After cooling, the powder obtained was ball-milled uniformly. The active materials (S@GeS_2_/rGO, S@MoS_2_/rGO and S@GeS_2_-MoS_2_/rGO) were mixed with conductive carbon black (Super P) and polyvinylidene fluoride (PVDF) (8 : 1 : 1 by mass) in *N*-methyl-2-pyrrolidone (NMP, 99.5%) solution to prepare the working electrodes. The prepared homogeneous slurry was coated on a piece of aluminum foil and vacuum dried at 60 °C overnight. The diameter of each composite cathode was 12 mm and the average surface loading was 2.0 mg cm^−2^. And pieces of lithium foil were used as the anodes and Celgard 2400 films were used as separators to assemble coin-type (LIR2032) batteries. The electrolyte was 1.0 M lithium bis(trifluoromethanesulfonyl) imide (LiTFSI, 99%) in a solvent mixture of 1,2-dimethoxyethane (DME) and 1,3-dioxolane (DOL) (1 : 1 by volume) with 2 wt% LiNO_3_. The coin batteries were assembled in an Ar-filled glove box (H_2_O and O_2_ < 1.0 ppm) and 40 μL mg^−1^ of electrolyte was used for each battery (the electrolyte-to-sulfur ratio was 11.8 μL mg^−1^).

### 4.3 Li_2_S_6_ adsorption test

The adsorption experiment was carried out in a glove box fitted with an Ar atmosphere. Li_2_S_6_ solution was prepared by mixing sulfur powder with Li_2_S (99.9%, Alfa Aesar) in a 5 : 1 molar ratio, which was dissolved in DOL and DME (1 : 1 volume ratio) solution and stirred for 12 h. After that, 20 mg of samples (GeS_2_/rGO, MoS_2_/rGO and GeS_2_-GeS_2_-MoS_2_/rGO) were added to the Li_2_S_6_ solution (2 mM, 4 mL) and left for several hours. After 12 h, the liquid supernatant from the bottle was extracted as a sample for ultraviolet-visible (UV-vis) testing.

### 4.4 Li_2_S nucleation test

Li_2_S_8_ catholyte was prepared by mixing sulfur powder and Li_2_S (7 : 1 molar ratio) and dissolved in tetraglyme solution and stirred overnight. Different samples (GeS_2_/rGO, MoS_2_/rGO and GeS_2_-MoS_2_/rGO) were prepared as working electrodes, and pieces of Li foil as counter electrodes. And the coin-type batteries were assembled with a Celgard 2400 membrane as the separator. 20 μL of Li_2_S_8_ catholyte was added into the cathode side, and 20 μL of the above electrolyte without Li_2_S_8_ was used as the anolyte drop to the anode side. The batteries were first galvanostatically discharged to 2.06 V at 0.012 mA, then discharged potentiostatically at 2.05 V until the current was below 0.01 mA. The nucleation rate and the specific capacity of Li_2_S deposition were evaluated by Faraday's law.^59^ After the nucleation test, the cathodes were disassembled and washed in a tetraglyme solution in an Ar-filled glove box to observe the morphology of Li_2_S. Theoretical equations of the current–time transients of four classic electrochemical deposition models (2D instantaneous nucleation (eqn (4)) and 2D progressive nucleation (eqn (5)) are based on Bewick, Fleischman, and Thirsk models; 3D instantaneous nucleation (eqn (6)) and 3D progressive nucleation (eqn (6)) are based on Scharifker–Hills models):4
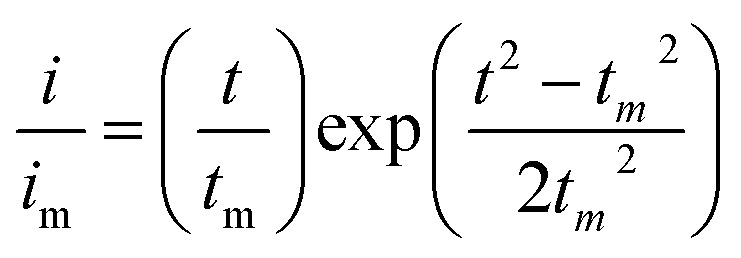
5
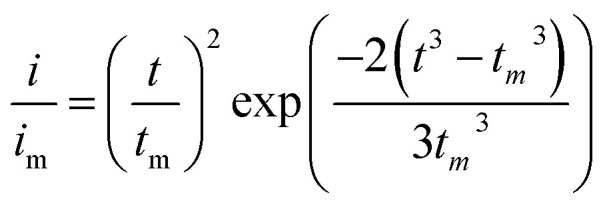
6
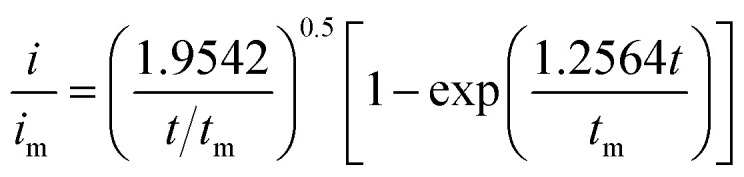
7
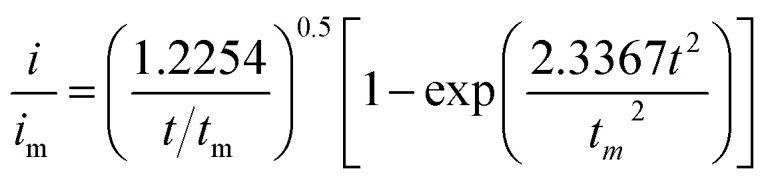
where *i* and *t* are the current density and time. And *i*_m_ and *t*_m_ are the maximum current density and the time at which the maximum current density occurs, respectively.

### 4.5 Li_2_S dissolution test

The dissolution of Li_2_S on the three electrodes was tested in an assembled CR2032 coin cell, where the different electrodes were used as working electrodes and lithium foil was used as the counter electrode. A 20 μL solution of 2.0 mol L^−1^ Li_2_S_8_ and 1.0 mol L^−1^ LiTFSI in tetraglyme was applied as catholyte, and 20 μL of control electrolyte without Li_2_S_8_ was used as anolyte. The above assembled batteries were first galvanostatically discharged to 1.80 V at 0.1 mA and then followed by 1.80 V at 0.01 mA, so that LiPSs were completely converted to solid Li_2_S. Then, the batteries were charged potentiostatically at 2.40 V until the current was below 0.01 mA for the oxidization process from solid Li_2_S to liquid LiPSs.

### 4.6 Symmetric battery test

Symmetric batteries were assembled in the same way as LSBs. Two electrodes with the same active materials (GeS_2_/rGO, MoS_2_/rGO, or GeS_2_-GeS_2_-MoS_2_/rGO) served as the working and counter electrodes. 40 μL of 0.5 M Li_2_S_6_ solution prepared by the adsorption test was used as the electrolyte. They were assembled into a typical CR2032 coin cell with a polypropylene (PP) membrane as the separator. For comparison, symmetric batteries with GeS_2_-MoS_2_/rGO electrodes and the above solution without Li_2_S_6_ were also assembled and tested. CV curves were obtained by using an electrochemical workstation in a voltage window of −1.0 to 1.0 V at a scan rate of 20 mV s^−1^.

### 4.7 Theoretical calculations

Density functional theory (DFT) calculations were performed using the projector-augmented wave pseudopotentials (PAW) in the Vienna *ab initio* simulation package (VASP) software.^60,61^ The generalized gradient approximation (GGA) with the Perdew–Burke–Ernzerhof (PBE) function was employed to handle the exchange-correlation energy. The weak intermolecular interactions between atoms are finely described by the DFT-D3 correction method in Grimme's scheme.^62^ The plane-wave basis set with a cutoff energy of 500 eV was set. Heterointerfaces were built at a relatively low crystal parameter mismatch (less than 5.0%).^63^ In the vertical direction, a 20 Å vacuum layer was established for all surfaces regardless of the periodic layer effect. A *k*-point grid of 1 × 1 × 1 determined by the Gamma-centered Monkhorst–Pack method in the Brillouin zone was used for GeS_2_ (311), MoS_2_ (002), and the mixed heterostructure, respectively. The geometry optimization was considered convergent when the force change was below 0.02 eV Å^−1^. The electron energy was considered self-consistent when the energy change was less than 10^−5^ eV. The U correction was adopted for the Mo atom in this system. The adsorption energies (*E*_ad_) of LiPSs were calculated by using the following equation:^64,65^

8 *E*_ad_ = *E*_total_ − *E*_Li_2_S_*x*__ − *E*_sub_

where *E*_total_ and *E*_sub_ are the energies of systems with and without the adsorption of LiPSs. *E*_Li_2_S_*x*__ is the energy of Li_2_S_*x*_ (Li_2_S, Li_2_S_2_, Li_2_S_4_, Li_2_S_6_, Li_2_S_8_, and S_8_). Therefore, a more negative *E*_ad_ represents a stronger adsorption ability.

### 4.8 Pouch battery assembly and measurements

Both the S@GeS_2_-MoS_2_/rGO cathode and lithium anode were cut into pieces (6 × 4 cm). The sulfur loading of the cathode in the pouch cell was 5.6 mg cm^−2^. The thickness of the lithium belt anode was 0.5 mm. And the *E*/*S* ratio was 6 μL mg^−1^. The separator (Celgard 2400) was sandwiched between the tailored S@GeS_2_-MoS_2_/rGO cathode and lithium anode. The electrochemical performances of pouch batteries are tested under the same conditions as those of coin cells. The energy density of the pouch battery is calculated using eqn (9):9
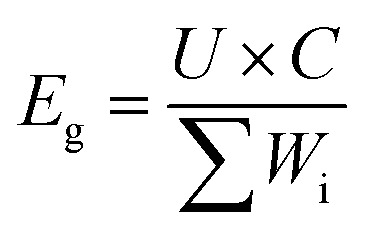
where *E*_g_ is the energy density, *U* is the average voltage (2.1 V), and *C* is the specific capacity of the cell. And *W*_i_ is the weight of individual battery components including sulfur cathodes, lithium anodes, electrolyte, separators, Al current collectors, and the battery package (total 6.057 g).

### 4.9 Material characterization

X-ray diffraction (XRD) patterns were recorded using an X-ray diffractometer (Rigaku D/max 2200 pc) with Cu (K_α_) radiation (*λ* = 1.54 Å) at 40 kV and 40 mA. For *in situ* XRD analysis, the *in situ* XRD cell was cycled at 1.7–2.8 V at 0.02C with a Neware battery test system. Thermogravimetric (TG) analysis was carried out by using a thermogravimetric analyzer (SHIMADZU, DTG-60AH) to obtain S loadings at a heating rate of 10 °C min^−1^ over a temperature range of 30 to 500 °C. Electronic conductivity of different samples was investigated using a CHI1140C workstation with a constant voltage of 1.0 V. All samples were pressed into compact discs and loaded into the cuvette for constant voltage testing, ultimately obtaining the parameters of the current over a period of time. XANES and EXAFS data reduction and analysis were processed using Athena software. The nitrogen adsorption isotherms were obtained by the Brunauer–Emmett–Teller method (BET, BSD-PM1/2, BSDINSTRU MENT). An ICP-OES (Agilent 5110) tester is used to analyze the Mo and Ge amounts in the GeS_2_-MoS_2_/rGO heterostructure. Atomic force microscopy (AFM) (Dimension Icon, Bruker, USA) was used to characterize the particle size and thickness distribution of GeS_2_-MoS_2_/rGO. The ultraviolet-visible (UV-vis) absorption spectra were measured in the range of 350–700 nm on a PerkinElmer Lambda 750 spectrophotometer. X-ray photoelectron spectroscopy (XPS) was conducted using a Thermo Scientific instrument (ESCALAB 250XI) with Al (K_α_) radiation. Scanning electron microscopy (SEM) images were collected with a field emission scanning electron microscope (JEOL JSM-7800F, 5/10 kV). Transmission electron microscopy (TEM), energy dispersive X-ray (EDX) and high resolution transmission electron microscopy (HRTEM) images were observed on a Tecnai G2F20 TWIN and JEM-2100F.

### 4.10 Electrochemical characterization

The galvanostatic discharge/charge was tested on a LAND battery tester (1.7–2.8 V). Cyclic voltammetry (CV) measurements were conducted and electrochemical impedance spectroscopy (EIS) data were obtained using a CHI 660D workstation (scan rate of 0.1 mV s^−1^) and a Princeton 1260A impedance analyzer (amplitude of 10 mV and frequency range of 10^−2^ to 10^5^ Hz), respectively. All tests were conducted at room temperature. The capacity was calculated based on the mass of sulfur.

## Data availability

Data are available from the authors upon reasonable request.

## Author contributions

Xun Jiao: writing the original draft, methodology, investigation. Xiaoxia Tang: methodology, investigation. Jinrui Li: methodology. Yujiao Xiang: investigation. Cunpu Li: data curation, formal analysis, funding acquisition, project administration, writing–review & editing, conceptualization, supervision. Cheng Tong: data curation, formal analysis, funding acquisition, project administration, writing–review & editing, conceptualization, supervision. Minhua Shao: writing–review & editing. Zidong Wei: data curation, project administration, formal analysis, writing–review & editing.

## Conflicts of interest

The authors declare that they have no known competing financial interests or personal relationships that could have appeared to influence the work reported in this paper.

## Supplementary Material

SC-015-D4SC02420F-s001
