# Correction: Toward robust lithium–sulfur batteries *via* advancing Li_2_S deposition

**DOI:** 10.1039/d4sc90096k

**Published:** 2024-05-31

**Authors:** Xun Jiao, Xiaoxia Tang, Jinrui Li, Yujiao Xiang, Cunpu Li, Cheng Tong, Minhua Shao, Zidong Wei

**Affiliations:** a State Key Laboratory of Advanced Chemical Power Sources, School of Chemistry and Chemical Engineering, Chongqing University Chongqing China lcp@cqu.edu.cn tongcheng@cqu.edu.cn zdwei@cqu.edu.cn; b Department of Chemical and Biological Engineering, The Hong Kong University of Science and Technology Clear Water Bay Kowloon Hong Kong

## Abstract

Correction for ‘Toward robust lithium–sulfur batteries *via* advancing Li_2_S deposition’ by Xun Jiao *et al.*, *Chem. Sci.*, 2024, https://doi.org/10.1039/D4SC02420F.

The authors regret that the affiliations were incomplete in the original manuscript. The corrected list of affiliations for this paper is as shown here.

The Royal Society of Chemistry apologises for these errors and any consequent inconvenience to authors and readers.

## Supplementary Material

