# Supplementary material for: Toward robust lithium–sulfur batteries via advancing Li2S deposition
Source: Chem Sci. 2024 May 1;15(21):7949–64. doi: 10.1039/d4sc02420f (PMC11134335; doi:10.1039/d4sc02420f)
Supplement: SC-015-D4SC02420F-s001 [file SC-015-D4SC02420F-s001.pdf]

Electronic Supplementary Material (ESI) for Chemical Science.
This journal is © The Royal Society of Chemistry 2024

Supplementary Information

Toward Robust Lithium-Sulfur Battery *via* Advancing Li_2S Deposition

Xun Jiao[a], Xiaoxia Tang[a], Jinrui Li[a], Yujiao Xiang[a], Cunpu Li[*,a], Cheng Tong[*,a], Minhua Shao[b], Zidong Wei[*,a]

[a] State Key Laboratory of Advanced Chemical Power Sources, School of Chemistry and Chemical Engineering, Chongqing University, Chongqing, 400044, China.

[b] Department of Chemical and Biological Engineering, The Hong Kong University of Science and Technology, Clear Water Bay, Kowloon, Hong Kong, China.

E-mail: lcp@cqu.edu.cn; tongcheng@cqu.edu.cn; zdwei@cqu.edu.cn

1. Supplementary figures

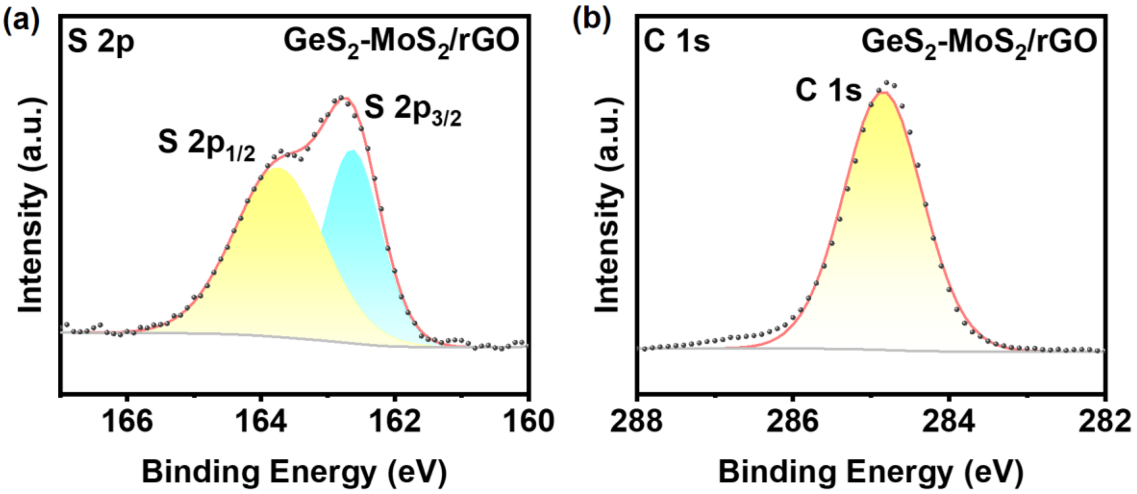

Figure S1 (a-b) High-resolution S 2p and C 1s XPS spectra of GeS_2-MoS_2/rGO, respectively.

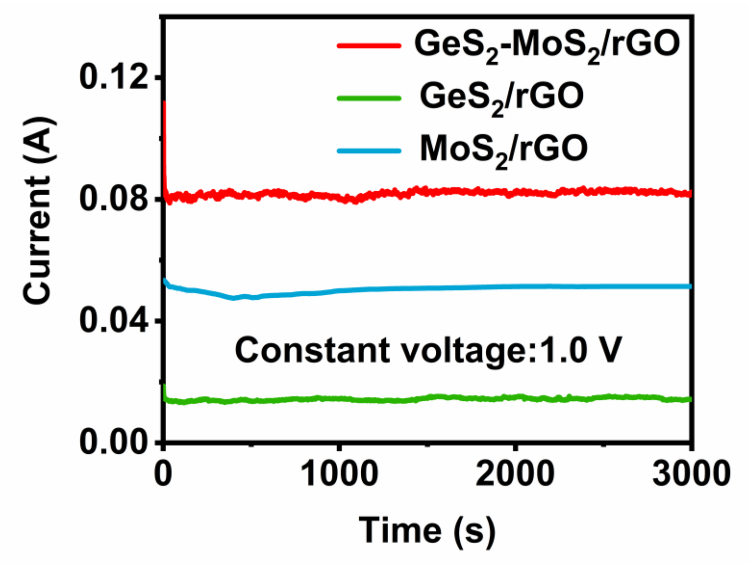

Figure S2 Electronic conductivity of GeS_2-MoS_2/rGO heterostructure, GeS_2/rGO, and MoS_2/rGO under constant voltage of 1.0 V.

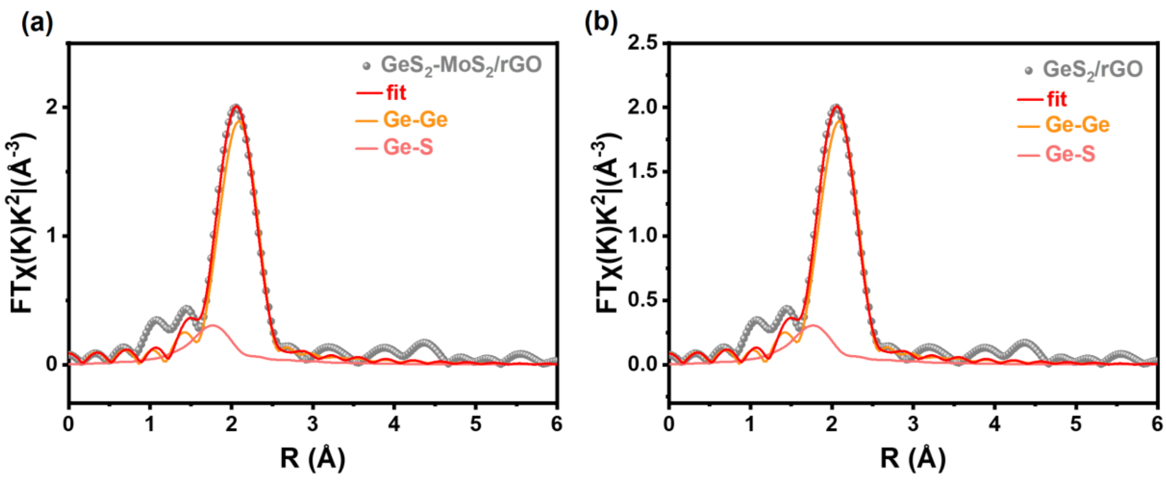

Figure S3 (a-b) The fitted R-space of EXAFS analysis of Ge in GeS_2-MoS_2/rGO and GeS_2/rGO, respectively.

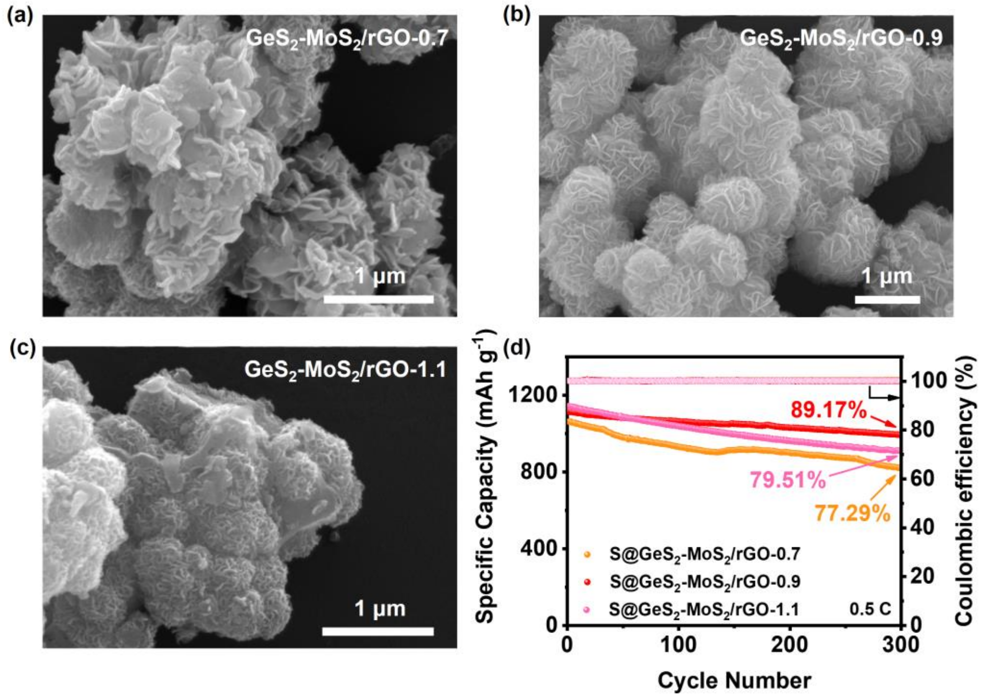

Figure S4 (a-c) SEM images of GeS_2-MoS_2/rGO with different ratios; (d) Cycling capacity of GeS_2-MoS_2/rGO with different ratios at 0.5 C over 300 cycles.

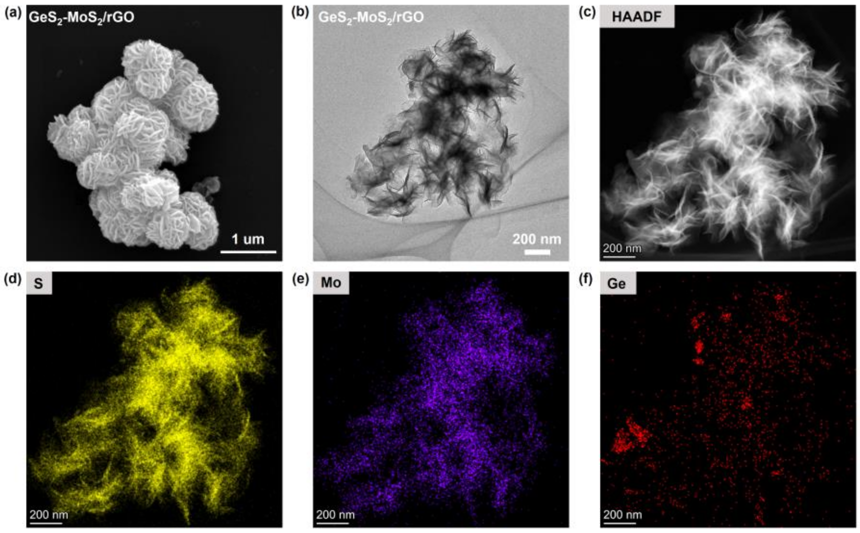

Figure S5 (a) SEM image of GeS_2-MoS_2/rGO; (b) TEM image of GeS_2-MoS_2/rGO; (c-f) EDX images of GeS_2-MoS_2/rGO.

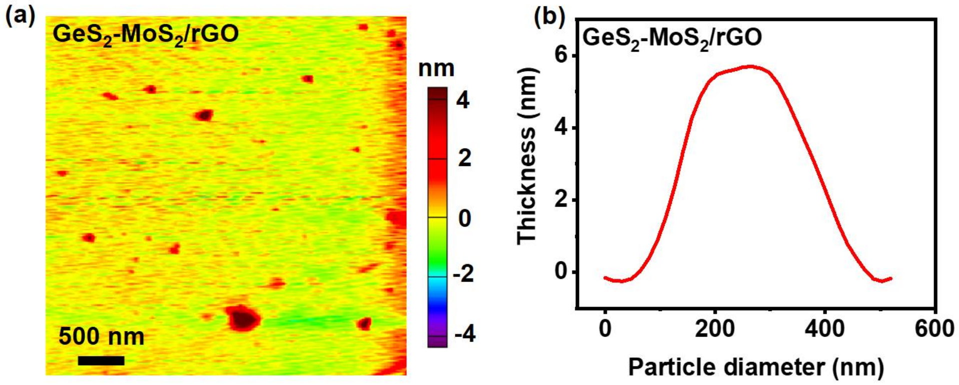

Figure S6 (a) AFM image and corresponding (b) particle size and thickness distribution of GeS_2-MoS_2/rGO. The particle distribution of GeS_2-MoS_2/rGO is 100-500 nm with a thickness of about 6 nm.

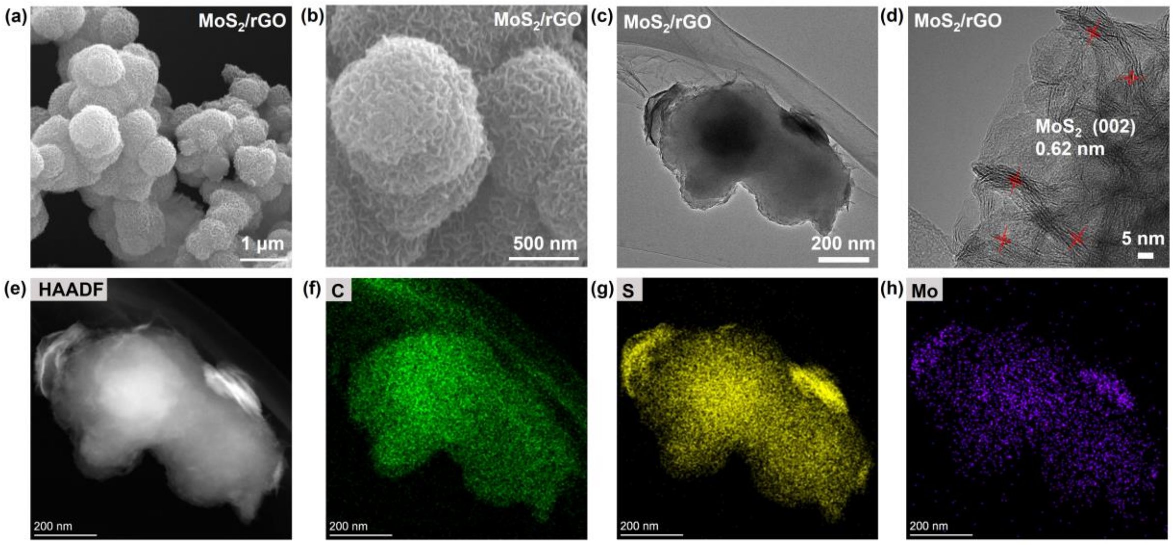

Figure S7 (a-b) SEM images of the MoS_2/rGO; (c-d) TEM and HRTEM images of the MoS_2/rGO; (e-h) EDX images of the MoS_2/rGO.

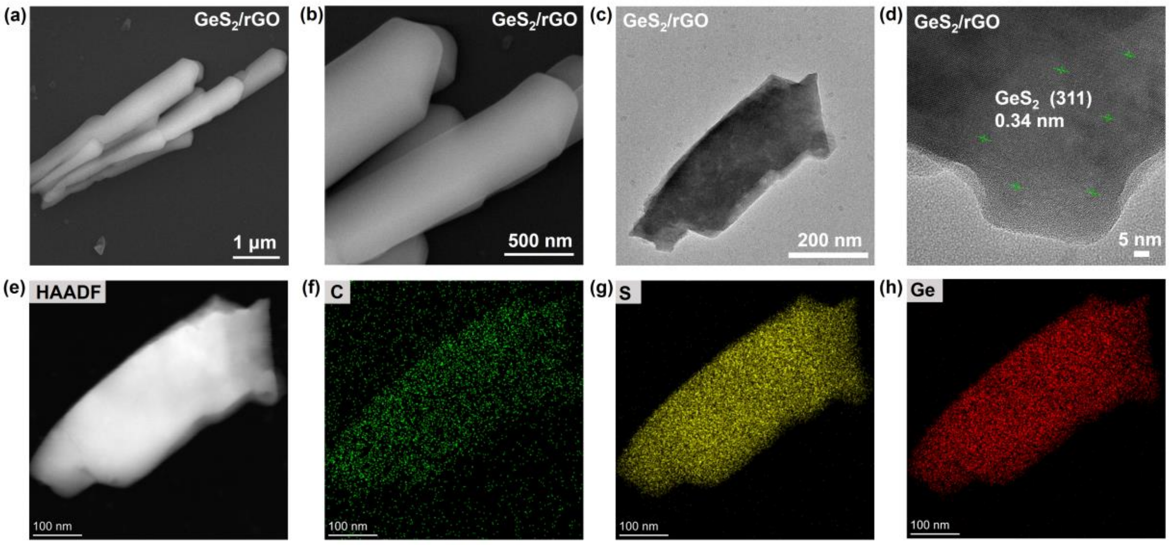

Figure S8 (a-b) SEM images of the GeS_2/rGO; (c-d) TEM and HRTEM images of the GeS_2/rGO; (e-h) EDX images of the GeS_2/rGO.

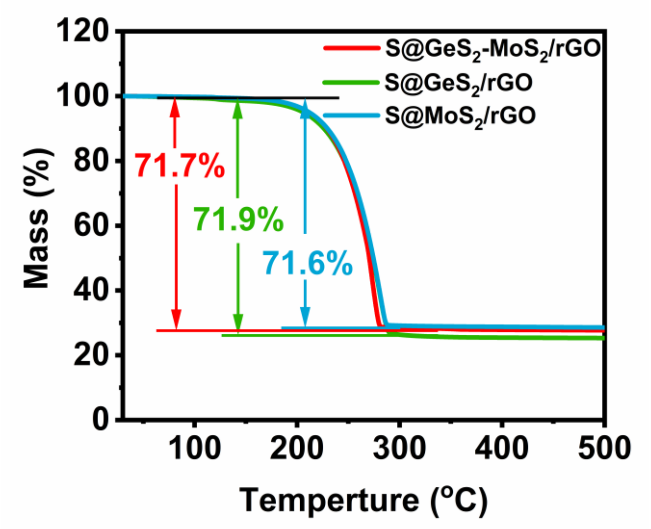

Figure S9 TG analyses of S@GeS_2-MoS_2/rGO, S@GeS_2/rGO and S@MoS_2/rGO.

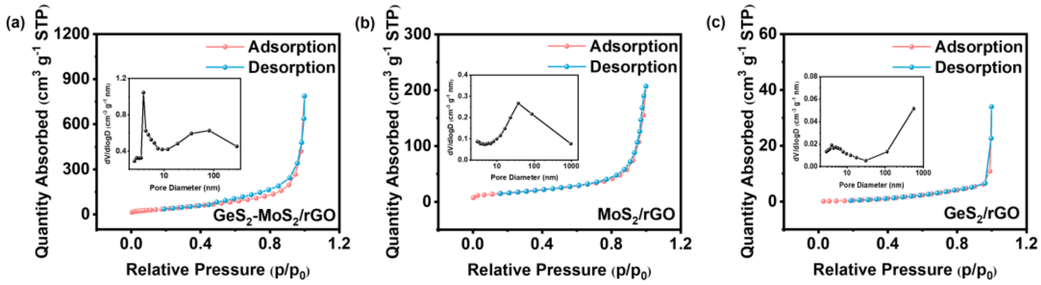

Figure S10 (a-c) BET patterns of GeS_2-MoS_2/rGO, MoS_2/rGO and GeS_2/rGO.

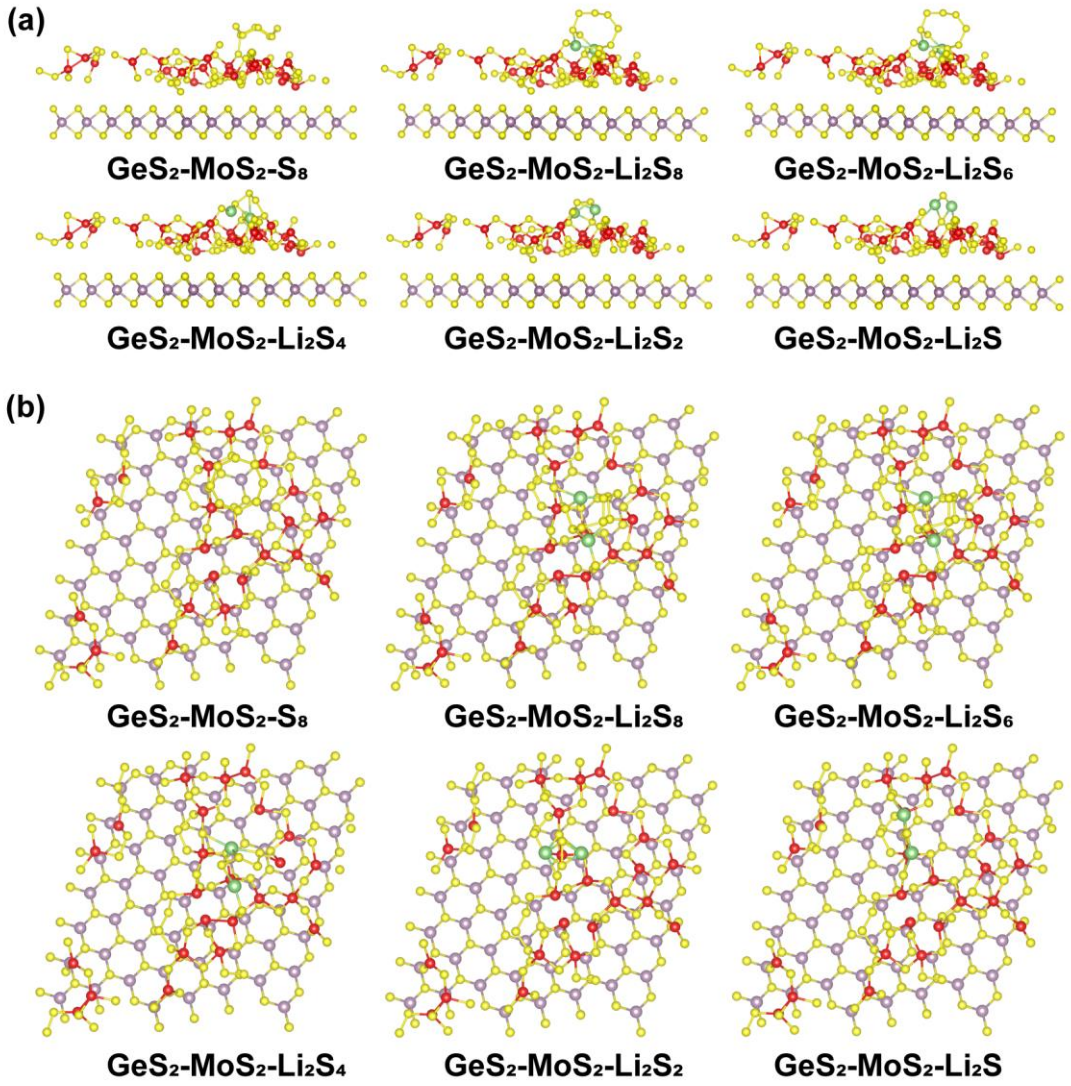

Figure S11 Side (a) and top (b) views of the adsorption configurations of LiPSs on the GeS_2-MoS_2 heterostructure.

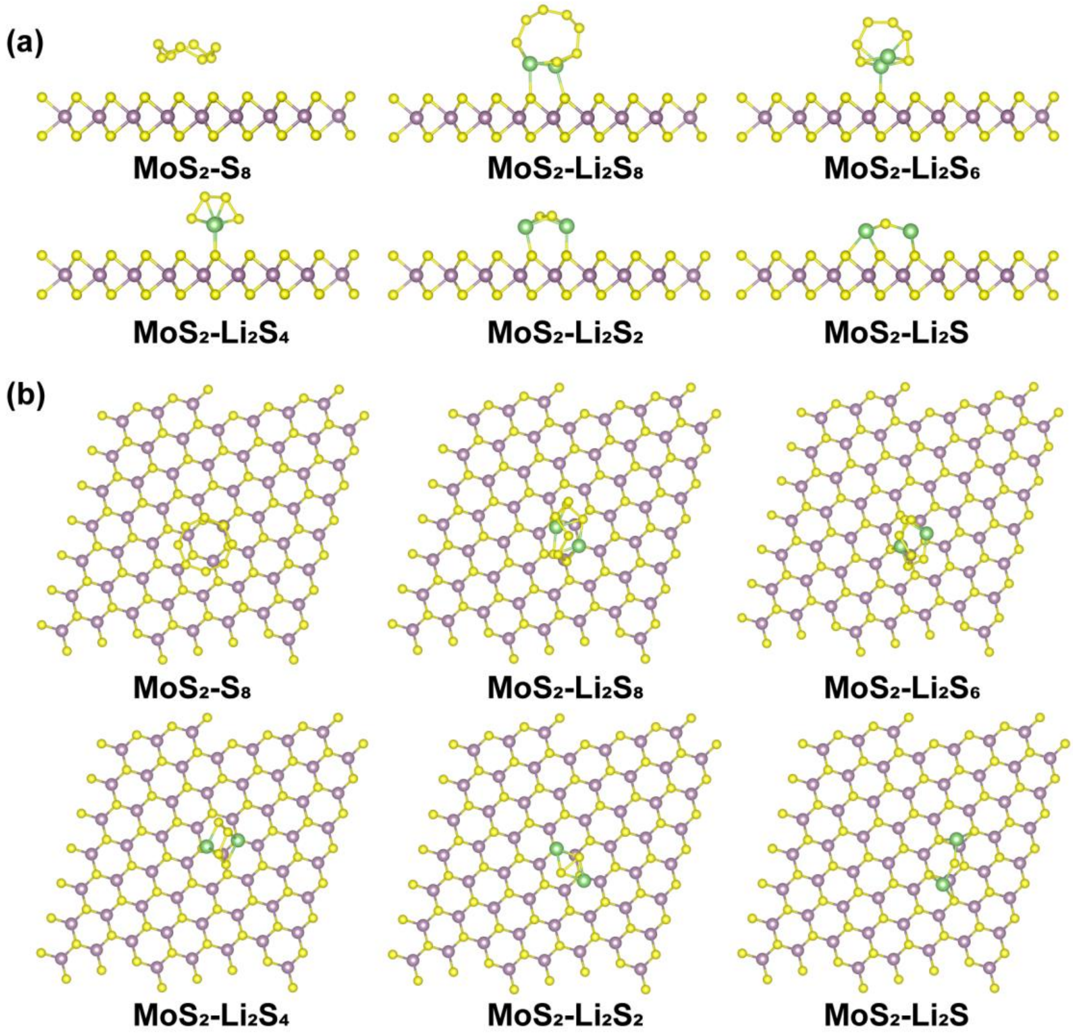

Figure S12 Side (a) and top (b) views of the adsorption configurations of LiPSs on MoS_2 (002).

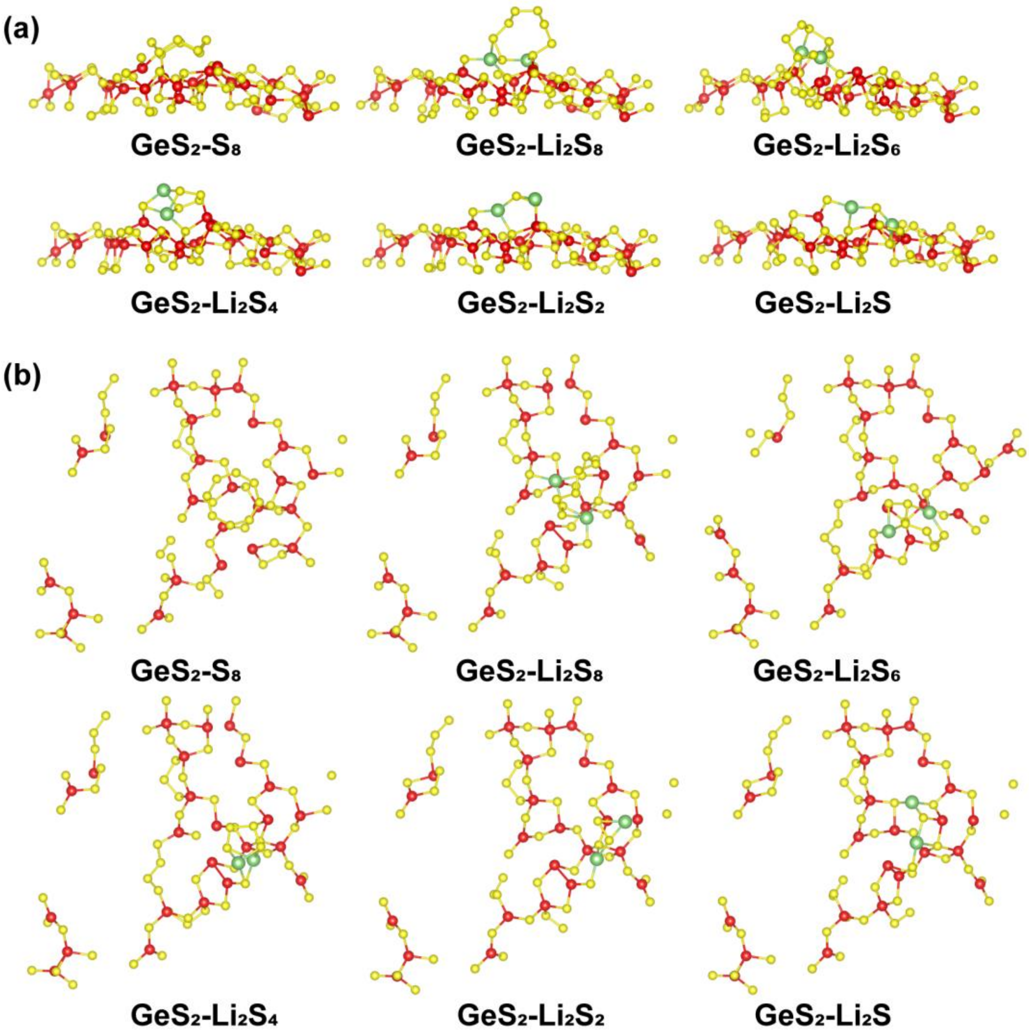

Figure S13 Side (a) and top (b) views of the adsorption configurations of LiPSs on GeS_2 (311).

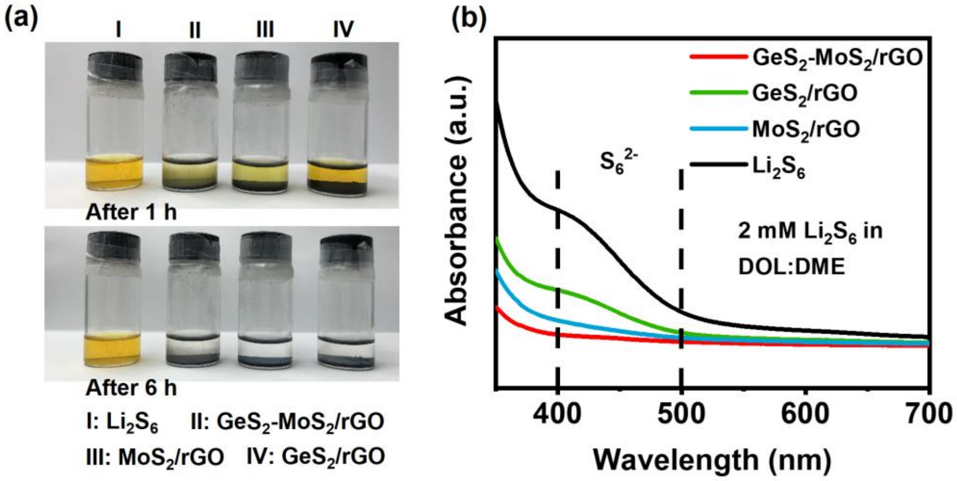

Figure S14 (a) Optical images of Li_2S_6 solutions after 1 h and 6 h adsorption, respectively; (b) UV-Vis spectra of Li_2S_6 solutions after exposure to different catalysts.

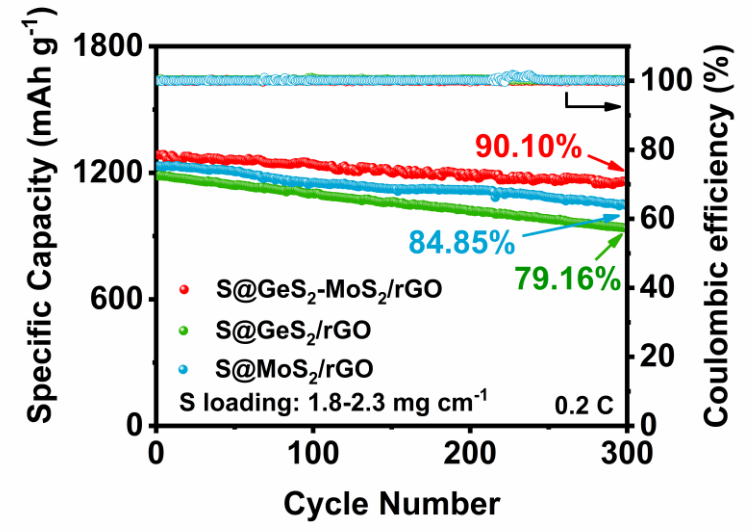

Figure S15 Cycling life of different electrodes at 0.2 C over 300 cycles.

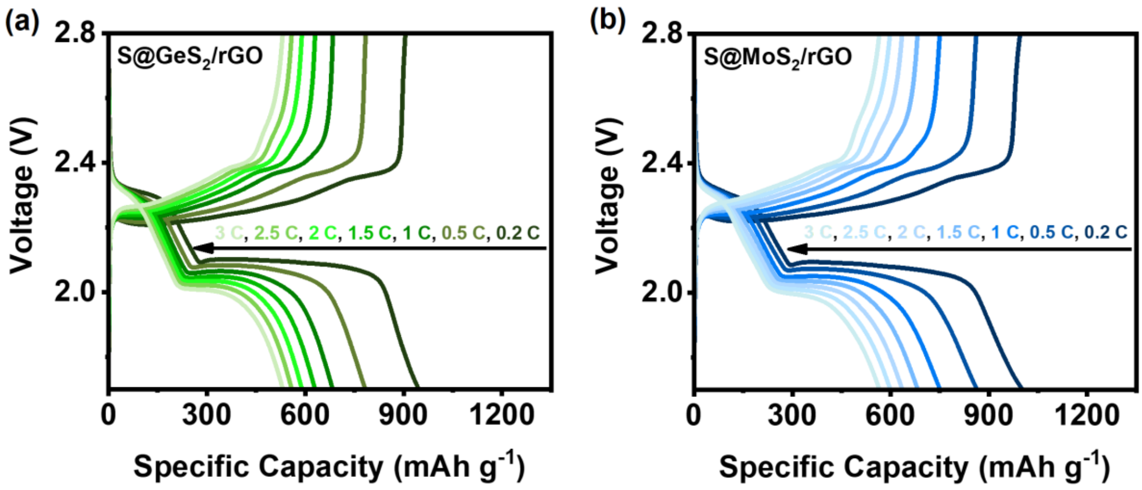

Figure S16 (a-b) Galvanostatic discharge/charge profiles of S@GeS_2/rGO and S@MoS_2/rGO with various current densities, respectively.

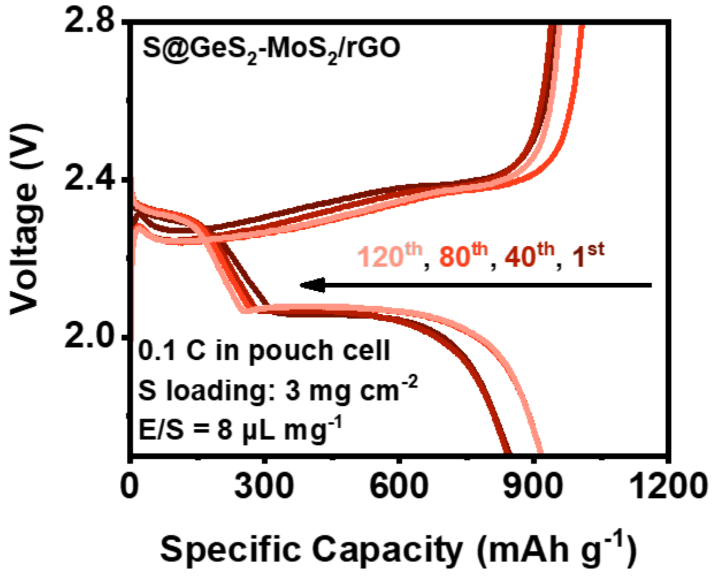

Figure S17 Galvanostatic discharge/charge profiles of pouch cell with the S@GeS_2-MoS_2/rGO cathode.

2. Supplementary tables

Table S1. The electronic conductivity (*σ*) values of different catalysts.

Catalyst	*I* (A)	*R* (1/S)	*L* (mm)	*σ* (S/mm)
GeS_2-MoS_2/rGO	0.0822	12.165	0.736	$4.56*10^{-4}$
GeS_2/rGO	0.0145	68.966	0.743	$8.12*10^{-5}$
MoS_2/rGO	0.0511	19.569	0.723	$2.78*10^{-4}$

Table S2. EXAFS fitting parameters at the Mo K-edge for various samples.

Sample	Shell	CN^a	R(Å)^b	σ^2(Å^2)^c	ΔE_0(eV)^d	R factor
Mo foil	Mo-Mo	8.0*	2.72 ±0.01	0.0034	4.7	0.0032
	Mo-Mo	6.0*	3.14 ±0.01	0.0030	6.7	
GeS_2-MoS_2/rGO	Mo-S	3.7 ±0.1	2.41 ±0.01	0.0037	4.0	0.0041
	Mo-Mo	2.7 ±0.2	3.14 ±0.01	0.0059	-1.8	
MoS_2/rGO	Mo-S	3.6 ±0.1	2.41 ±0.01	0.0025	4.0	0.0011
	Mo-Mo	2.5 ±0.1	3.15 ±0.01	0.0027	0.2	

^{a}*CN*, coordination number; ^{b}*R*, distance between absorber and backscatter atoms; $^c\sigma^2$, Debye-Waller factor to account for both thermal and structural disorders; $^d\Delta E_0$, inner potential correction; *R* factor indicates the goodness of the fit. S_0^2 was fixed to 0.89. A reasonable range of EXAFS fitting parameters: $0.700 < S_0^2 < 1.000$; $CN > 0$; $\sigma^2 > 0$ Å^2; $|\Delta E_0| < 15$ eV; R factor < 0.02.

Table S3. EXAFS fitting parameters at the Ge K-edge for various samples.

Sample	Shell	CN^a	$R(Å)^b$	$\sigma^2(Å^2)^c$	$\Delta E_0(eV)^d$	R factor
Ge foil	Ge-Ge	4.0*	2.46 ±0.01	0.0049	4.7	0.0063
GeS_2-MoS_2/rGO	Ge–S	0.6 ±0.3	2.17 ±0.01	0.0089	13.6	0.0152
	Ge-Ge	3.6 ±0.3	2.40 ±0.01	0.0052	5.1	
GeS_2-rGO	Ge–S	0.8 ±0.3	2.03 ±0.01	0.0150	-5.5	0.0122
	Ge-Ge	3.7 ±0.2	2.40 ±0.01	0.0042	4.3	

^{a}*CN*, coordination number; ^{b}*R*, distance between absorber and backscatter atoms; $^c\sigma^2$, Debye-Waller factor to account for both thermal and structural disorders; $^d\Delta E_0$, inner potential correction; *R* factor indicates the goodness of the fit. S_0^2 was fixed to 0.96. A reasonable range of EXAFS fitting parameters: $0.700 < S_0^2 < 1.000$; $CN > 0$; $\sigma^2 > 0$ Å^2; $|\Delta E_0| < 15$ eV; R factor < 0.02.

Table S4. The element content of different ratios of MoS_2 and GeS_2 in the GeS_2-MoS_2/rGO heterostructure.

Ratio	Mo	Ge
MoS_2:GeS_2 = 0.7	15.15%	21.53%
MoS_2:GeS_2 = 0.9	20.18%	22.63%
MoS_2:GeS_2 = 1.1	22.09%	19.83%

Table S5. Impedance (R_s and R_{ct}) of host materials after cycling.

Electrode	R_s (Ω)	R_{ct} (Ω)
S@GeS_2-MoS_2/rGO	4.65	16.52
S@GeS_2/rGO	4.82	33.64
S@MoS_2/rGO	4.69	24.37

Table S6. The capacity retention of different cathodes.

Cathode	Discharge current (C)	Cycle number	Capacity retention	Reference
S/ZnSe-$CoSe_2$@NC	0.2	100th	79.97%	S9
	2	1000th	~63%	
W_2N/Mo_2N@MOF-C/S	0.5	260th	94.96%	S10
	1	980th	66.98%	
	3	2000th	60.8%	
La_2O_3-MXene@CNF/S	0.2	400th	85.2%	S11
	2	1000th	~68%	
MoS_2-MoN/S	0.2	100th	93.9%	S12
	2	1000th	59%	
**S@GeS_2-MoS_2/rGO**	0.2	300th	**90.1%**	
	0.5	300th	**89.17%**	***This work***
	3	1000th	**68.63%**	

3. Supplementary references

1. J. Xu, L. Xu, Z. Zhang, B. Sun, Y. Jin, Q. Jin, H. Liu and G. Wang, *Energy Storage Materials*, 2022, **47**, 223–234.

2. Y. Song, P. Tang, Y. Wang, Y. Wang, L. Bi, Q. Liang, L. He, Q. Xie, Y. Zhang, P. Dong, Y. Zhang, Y. Yao, J. Liao and S. Wang, *Journal of Energy Chemistry*, 2024, **88**, 363–372.

3. Z. Huang, Y. Zhu, Y. Kong, Z. Wang, K. He, J. Qin, Q. Zhang, C. Su, Y. Zhong and H. Chen, *Advanced Functional Materials*, 2023, **33**, 2303422.

4. S. Wang, S. Feng, J. Liang, Q. Su, F. Zhao, H. Song, M. Zheng, Q. Sun, Z. Song, X. Jia, J. Yang, Y. Li, J. Liao, R. Li and X. Sun, *Advanced Energy Materials*, 2021, **11**, 2003314.
